# Supplementary material for: Non-canonical autophagy drives alternative ATG8 conjugation to phosphatidylserine
Source: Mol Cell. 2021 May 6;81(9):2031–2040.e8. doi: 10.1016/j.molcel.2021.03.020 (PMC8122138; doi:10.1016/j.molcel.2021.03.020)
Supplement: Document S1. Figures S1–S4 [file mmc1.pdf]

Molecular Cell, Volume *81*

Supplemental information

Non-canonical autophagy drives alternative ATG8 conjugation to phosphatidylserine

Joanne Durgan, Alf H. Lystad, Katherine Sloan, Sven R. Carlsson, Michael I. Wilson, Elena Marcassa, Rachel Ulferts, Judith Webster, Andrea F. Lopez-Clavijo, Michael J. Wakelam, Rupert Beale, Anne Simonsen, David Oxley, and Oliver Florey

Supplemental Figure S1

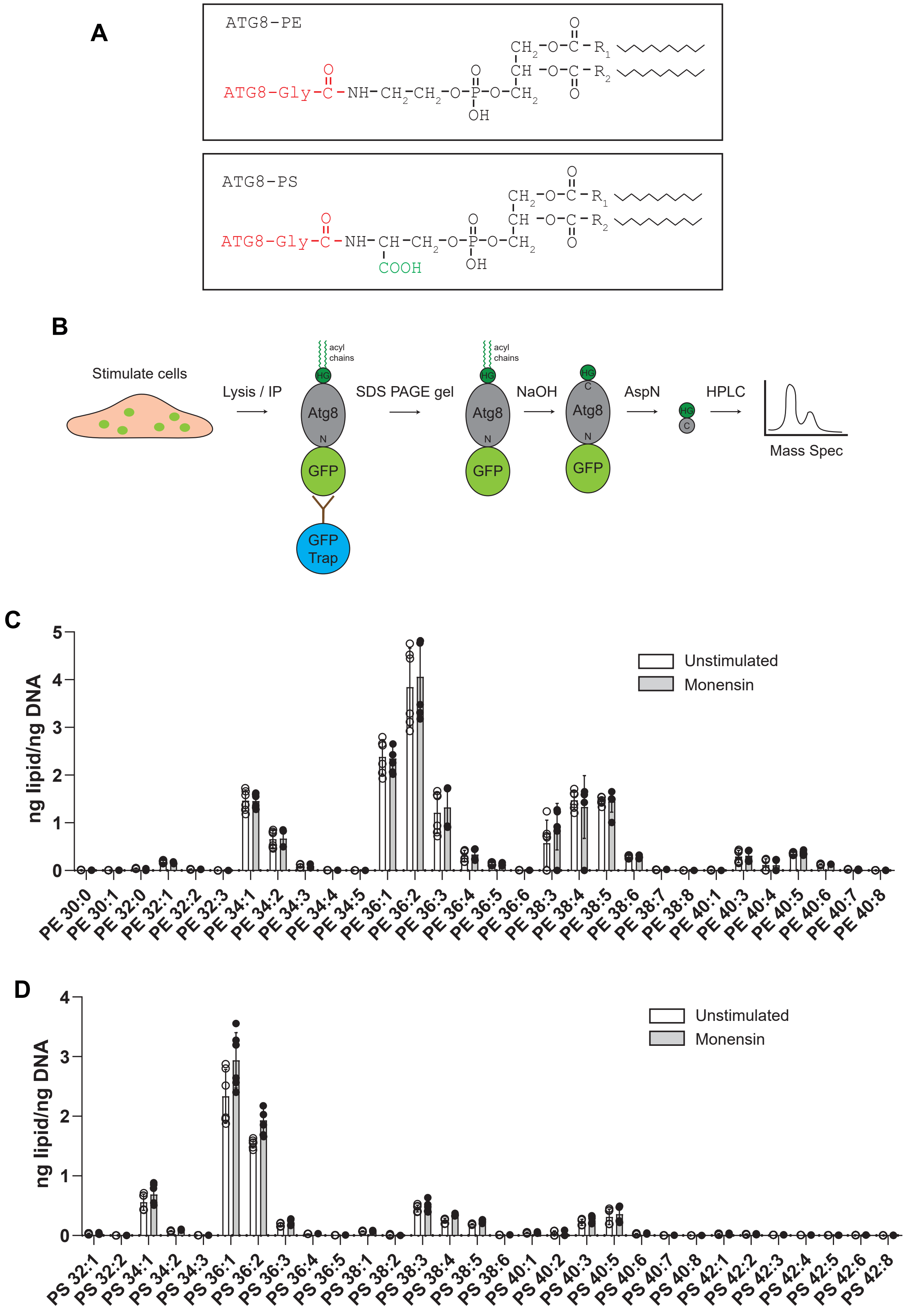

Figure S1. Analysis of ATG8 lipidation and PE/PS lipidomics, Related to Figure 1 and STAR Methods.

(A) Schematics representing ATG8 conjugated to PE and PS.
(B) Work flow for GFP-ATG8 mass spectrometry analysis. Cells are stimulated as indicated, then subjected to lysis and GFP-ATG8 immunoprecipitation using GFP-TRAP beads. Samples are run on SDS-PAGE gels, coomassie stained and appropriate bands excised and base treated (NaOH) to strip lipid acyl chains. HG = lipid headgroup. Samples are then digested using AspN protease, separated by HPLC and analysed by mass spectrometry.
(C and D) Global lipids quantification from *ATG13*$^{-/-}$ MCF10A cells -/+ 100 μM monensin, for 60 mins, were analysed for phosphoethanolamine (PE) (C) or phosphatidylserine (PS) (D) molecular species. Data represent means from 6 biological replicates ± SD, with no major changes.

Supplemental Figure S2

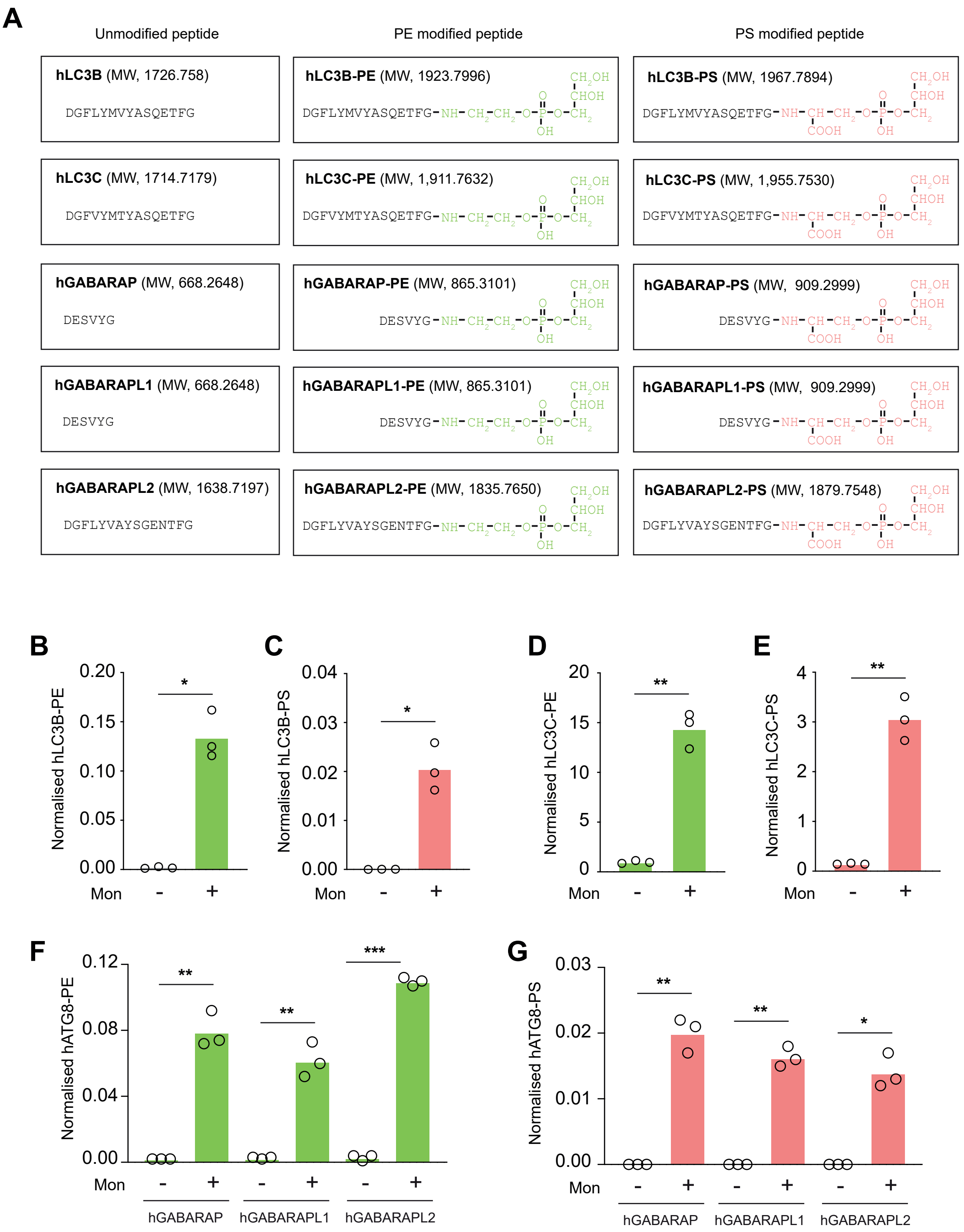

Figure S2. All human ATG8 proteins are conjugated to PS during non-canonical autophagy, Related to Figure 1.

(A) Schematics representing C-terminal peptides of human LC3B, LC3C, GABARAP, GABARAPL1 and GABARAPL2 proteins conjugated to either the PE or PS headgroup. Predicted MWs are indicated.

(B – G) Normalised mass spectrometry analysis of GFP-tagged human ATG8 proteins from monensin treated MCF10A *ATG13*$^{-/-}$ cells.

Data represent means from 3 independent experiments, ***$p<0.0002$, **$p<0.002$, *$p<0.03$, paired t-test.

Supplemental Figure S3

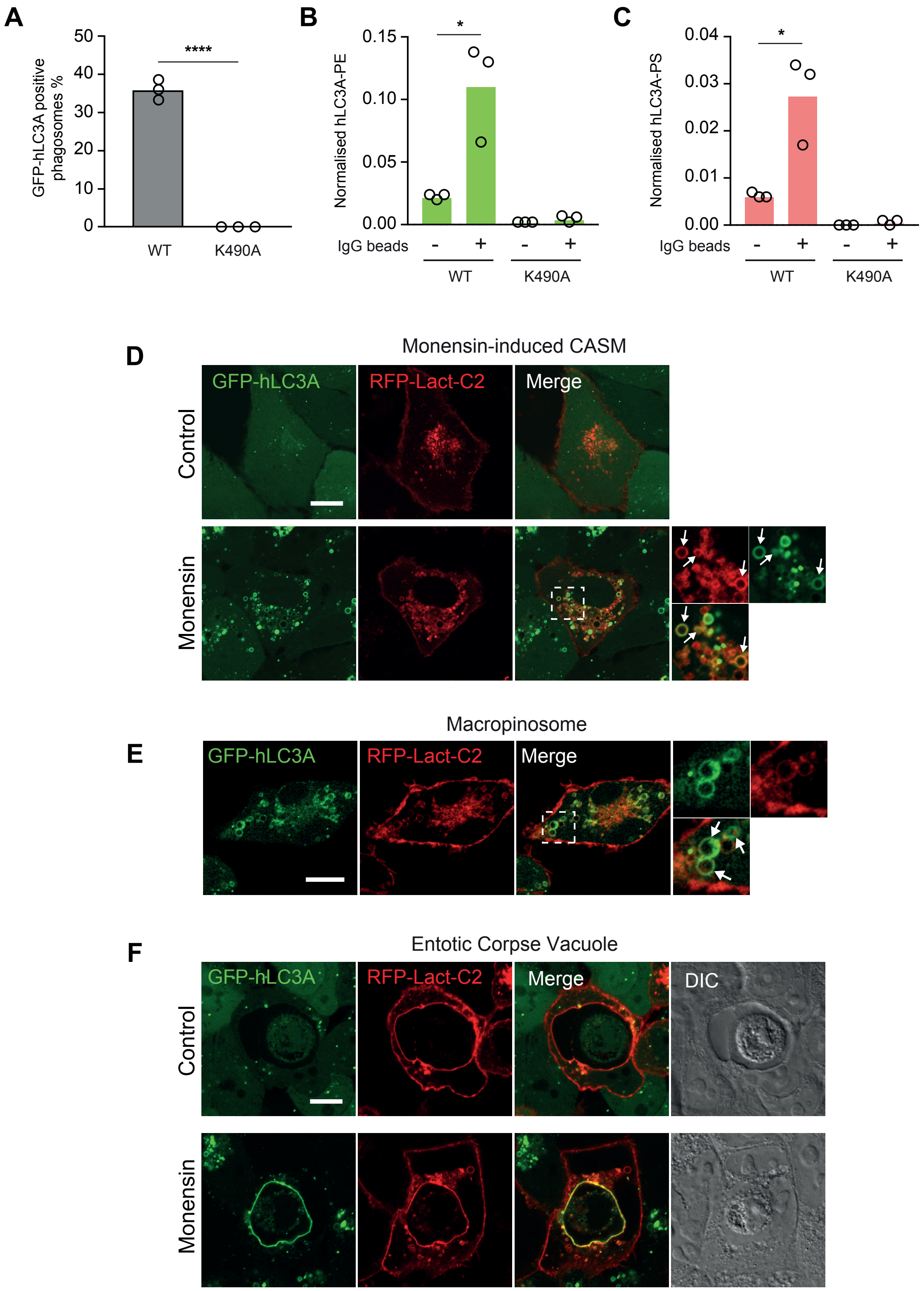

Figure S3. ATG8-PS conjugation depends on the ATG16L1 WD40 domain during LAP, and GFP-LC3A is recruited to PS positive membranes during CASM, Related to Figure 3.

(A) Quantification of GFP-hLC3A positive IgG-bead containing phagosomes in RAW264.7 ATG16L1-/- cells re-expressing WT or K490A ATG16L1. Data represent means from 3 independent experiments, WT n=189, K490A n=150 phagosomes in total, ****$p<0.0001$, unpaired t-test.

(B and C) Normalised mass spectrometry analysis of GFP-hLC3A conjugated to PE or PS during LAP in RAW264.7 cells expressing ATG16L1 WT or K490A. Data represent means from 3 independent experiments, *$p<0.01$, unpaired t-test.

(D) Confocal images of MCF10A expressing GFP-hLC3A and RFP-Lact-C2 +/- monensin treatment for 40 mins. Dashed box indicates zoomed region. Arrows indicate double-positive membranes. Scale bar: 10 μm.

(E) Confocal images of J774A.1 cells expressing GFP-hLC3A and PS sensor RFP-Lact-C2. Arrows denote macropinosomes. Scale bar: 5 μm.

(F) Confocal images of entotic corpse vacuoles in MCF10A expressing GFP-hLC3A and RFP-Lact-C2 +/- monensin treatment. Scale bar: 10 μm.

Supplementary Figure S4

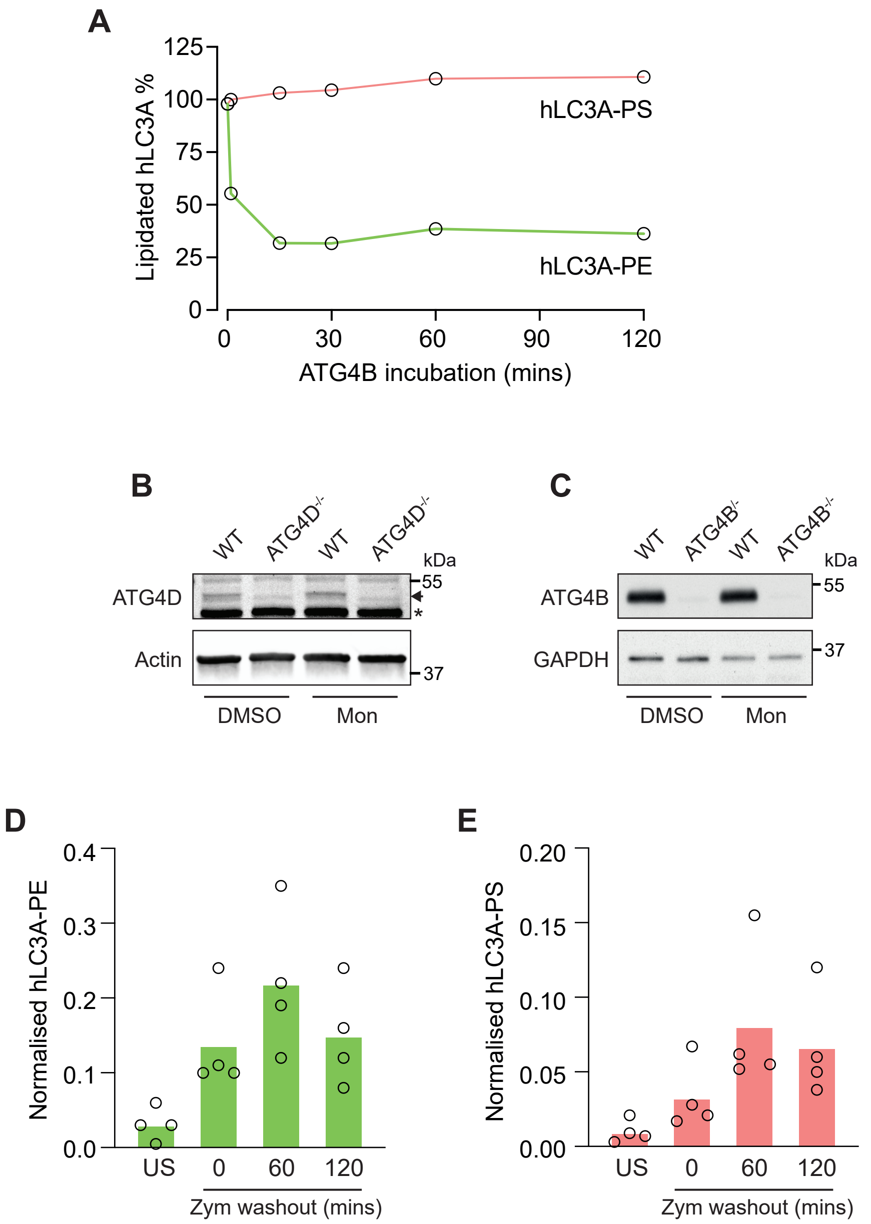

Figure S4. ATG4 delipidation of ATG8-PE and ATG8-PS, Related to Figure 4.

(A) Mass spectrometry analysis of GFP-hLC3A immunoprecipitated from MCF10A cells treated with monensin followed by incubation with recombinant ATG4B for the indicated times. Data are normalised to time 0.

(B and C) Western blot analysis of wild type and *ATG4D*$^{-/-}$ HCT116 cells (B) and wild type and *ATG4B*$^{-/-}$ HeLa cells (C) treated -/+ monensin. Arrow indicates ATG4D, asterisk indicates non-specific band.

(D and E) Normalized mass spectrometry analysis of GFP-hLC3A-PE and GFP-hLC3A-PS in RAW264.7 cells stimulated with zymosan for 25 mins followed by washout for the indicated times. Data represent means of 4 independent experiments.
