# Supplementary material for: Non-canonical autophagy drives alternative ATG8 conjugation to phosphatidylserine
Source: Mol Cell. 2021 May 6;81(9):2031–2040.e8. doi: 10.1016/j.molcel.2021.03.020 (PMC8122138; doi:10.1016/j.molcel.2021.03.020)
Supplement: Document S2. Article plus supplemental information [file mmc2.pdf]

Molecular Cell

Short Article

Non-canonical autophagy drives alternative ATG8 conjugation to phosphatidylserine

Graphical abstract

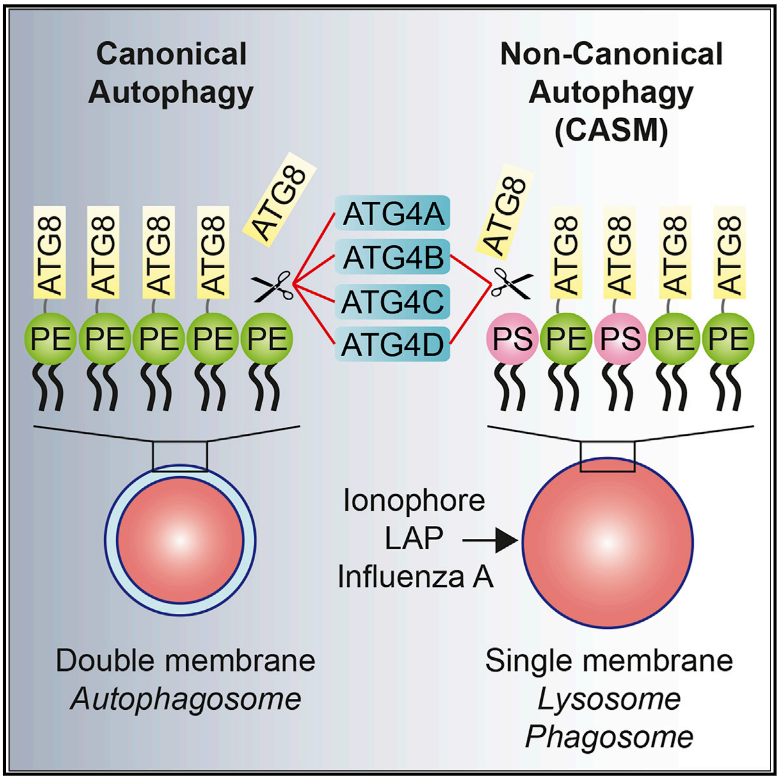

Highlights

- ATG8 can undergo alternative conjugation to phosphatidylserine in cells
- ATG8-PS occurs during non-canonical autophagy via single-membrane ATG8 conjugation
- ATG8-PS can be induced by LAP, influenza A, and lysosomal ionic imbalance
- ATG8-PS undergoes differential delipidation by ATG4 isoforms

Authors

Joanne Durgan, Alf H. Lystad, Katherine Sloan, ..., Anne Simonsen, David Oxley, Oliver Florey

Correspondence

oliver.florey@babraham.ac.uk

In brief

ATG8 conjugation to phosphatidylethanolamine is a hallmark feature of autophagy. Durgan et al. discover that ATG8 can undergo alternative conjugation, to phosphatidylserine, during non-canonical autophagy processes, such as phagocytosis, on single-membrane compartments. ATG8-PS and ATG8-PE bear different dynamics and are differentially regulated by ATG4 isoforms.

Durgan et al., 2021, Molecular Cell *81*, 2031–2040
May 6, 2021 © 2021 The Author(s). Published by Elsevier Inc.
https://doi.org/10.1016/j.molcel.2021.03.020

CellPress

Molecular Cell

CellPress
OPEN ACCESS

Short Article

Non-canonical autophagy drives alternative ATG8 conjugation to phosphatidylserine

Joanne Durgan,[1] Alf H. Lystad,[2,7] Katherine Sloan,[1,7] Sven R. Carlsson,[3] Michael I. Wilson,[1] Elena Marcassa,[4] Rachel Ulferts,[4] Judith Webster,[5] Andrea F. Lopez-Clavijo,[6] Michael J. Wakelam,[1,6] Rupert Beale,[4] Anne Simonsen,[2] David Oxley,[5] and Oliver Florey[1,8,*]
[1]Signalling Programme, Babraham Institute, Cambridge, UK
[2]Department of Molecular Medicine, University of Oslo, Oslo, Norway
[3]Department of Medical Biochemistry and Biophysics, Umeå University, Umeå, Sweden
[4]Francis Crick Institute, London, UK
[5]Mass Spectrometry Facility, Babraham Institute, Cambridge, UK
[6]Lipidomics Facility, Babraham Institute, Cambridge, UK
[7]These authors contributed equally
[8]Lead contact
*Correspondence: oliver.florey@babraham.ac.uk
https://doi.org/10.1016/j.molcel.2021.03.020

SUMMARY

Autophagy is a fundamental catabolic process that uses a unique post-translational modification, the conjugation of ATG8 protein to phosphatidylethanolamine (PE). ATG8 lipidation also occurs during non-canonical autophagy, a parallel pathway involving conjugation of ATG8 to single membranes (CASM) at endolysosomal compartments, with key functions in immunity, vision, and neurobiology. It is widely assumed that CASM involves the same conjugation of ATG8 to PE, but this has not been formally tested. Here, we discover that all ATG8s can also undergo alternative lipidation to phosphatidylserine (PS) during CASM, induced pharmacologically, by LC3-associated phagocytosis or influenza A virus infection, in mammalian cells. Importantly, ATG8-PS and ATG8-PE adducts are differentially delipidated by the ATG4 family and bear different cellular dynamics, indicating significant molecular distinctions. These results provide important insights into autophagy signaling, revealing an alternative form of the hallmark ATG8 lipidation event. Furthermore, ATG8-PS provides a specific "molecular signature" for the non-canonical autophagy pathway.

INTRODUCTION

A defining feature of autophagy is the lipidation of ATG8, a family of ubiquitin-like proteins including mammalian LC3A/B/B2/C and GABARAP/L1/L2 (Johansen and Lamark, 2020; Mizushima, 2020). Nascent pro-ATG8 is first primed by a cysteine protease, ATG4, to expose a conserved aromatic-Gly motif at its C terminus (Tanida et al., 2004). A ubiquitin-like conjugation system, composed of ATG7, ATG3, and ATG16L1/12/5, then drives the covalent ligation of this glycine to a lipid, phosphatidylethanolamine (PE), through an amide bond to its headgroup (Figure S1A) (Ichimura et al., 2000; Kirisako et al., 2000). This unique post-translational modification recruits ATG8 to autophagosomal membranes, where it modulates cargo loading and maturation (Johansen and Lamark, 2020; Nguyen et al., 2016). The associated relocalization of ATG8s and the characteristic protein band-shift between unlipidated (ATG8-I) and lipidated (ATG8-II) forms are widely used to define and assay autophagy-related processes (Klionsky et al., 2016; Mizushima and Yoshimori, 2007).

A second phospholipid, phosphatidylserine (PS), also bears an amino group in its head moiety (Figure S1A), which can be conjugated to ATG8 *in vitro* (Sou et al., 2006). However, *in vivo*, ATG8 lipidation occurs exclusively to PE, in both yeast (Ichimura et al., 2000) and mammalian cells (Sou et al., 2006). The mechanism underlying cellular specificity is not fully understood, but physiological pH and phospholipid composition may prohibit alternative lipidation to PS (Nakatogawa et al., 2008; Oh-oka et al., 2008).

The autophagy machinery also mediates critical, parallel functions in other vital cellular processes (Galluzzi and Green, 2019). During "non-canonical autophagy," a subset of core ATG proteins (ATG7/3/12/5/16L1), but not the upstream regulators (FIP200/ULK/ATG13), target various endolysosomal compartments for conjugation of ATG8 to single membranes (CASM). LC3-associated phagocytosis (LAP) is an important example, where LC3 conjugation to phagosomes, housing pathogens or apoptotic debris, modulates the immune response (Sanjuan et al., 2007), inflammation (Henault et al., 2012; Martinez et al., 2015, 2016), antigen presentation (Fletcher et al., 2018; Ma et al., 2012), vision (Kim et al., 2013), and tumor cell tolerance (Cunha et al., 2018). CASM is also active during macropinocytosis, entosis (Florey et al., 2011), LC3-associated endocytosis

This is an open access article under the CC BY license (http://creativecommons.org/licenses/by/4.0/).

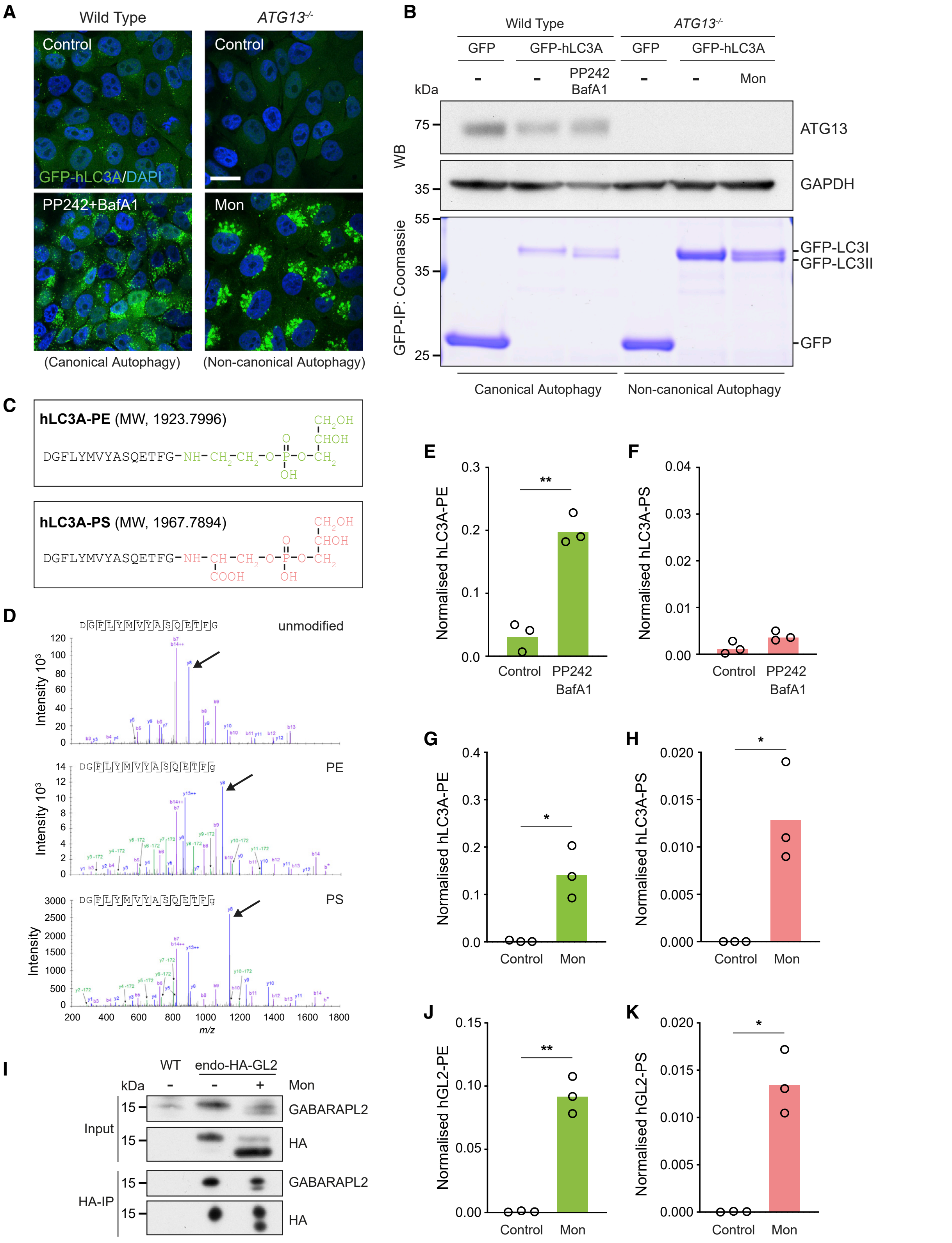

Figure 1. Pharmacological activation of non-canonical autophagy promotes ATG8-PS lipidation in cells

(A) Confocal images of WT and *ATG13*$^{-/-}$ MCF10A cells upon activation of canonical (PP242/BafA1) and non-canonical autophagy (monensin). Scale bar: 20 μm.

(B) Coomassie staining of GFP-IPs and western blotting of cells treated as in (A).

(legend continued on next page)

(Heckmann et al., 2019), and cGAS-STING activation (Fischer et al., 2020). In each case, the non-canonical autophagy pathway drives ATG8 lipidation, which has been widely assumed to represent PE conjugation (Cunha et al., 2018; Florey et al., 2011; Martinez et al., 2015). However, the identity of the modified ATG8 has not been formally tested in this context.

RESULTS

Mass spectrometric analysis of ATG8 lipidation

To investigate ATG8 lipidation during CASM, we took a mass spectrometric approach. GFP-tagged ATG8s were expressed in different cell lines, treated with different stimuli, to drive ATG8 lipidation associated with either canonical autophagy or CASM (Figure 1). GFP-ATG8 was then immunoprecipitated, base-treated to remove phospholipid acyl chains (leaving only the headgroup conjugated), and subjected to proteolytic cleavage with AspN protease (Figure S1B). The resulting ATG8 C-terminal peptides, in their unmodified form, or covalently conjugated to a phospholipid headgroup, were analyzed by liquid chromatography-tandem mass spectrometry. Where linked to glycerophosphoethanolamine (from PE), this peptide has a mass of 1,923.7996; if conjugated to glycerophosphoserine (from PS), the expected mass would be 1,967.7894.

As proof of concept, canonical autophagy was induced in wild-type (WT) cells expressing GFP-hLC3A, by co-treatment with mTOR (PP242) and V-ATPase (BafA1) inhibitors, which induce and accumulate autophagosomes, respectively. As expected, GFP-hLC3A relocalizes to punctate autophagosomes upon PP242/BafA1 treatment (Figure 1A), and a faster migrating, lipidated band is observed by Coomassie staining (Figure 1B). By mass spectrometry, lipidation corresponds exclusively to the covalent conjugation of PE, with negligible PS detected (Figures 1C–1F). These findings are consistent with published work, in which activation of autophagy *in vivo* induces the selective conjugation of ATG8 to PE (Ichimura et al., 2000; Sou et al., 2006).

To investigate ATG8 lipidation during non-canonical autophagy, *ATG13*$^{-/-}$ cells, deficient in canonical autophagy, were treated with monensin, a known inducer of CASM (Jacquin et al., 2017). Consistent with previous work (Fletcher et al., 2018; Florey et al., 2015), these conditions yield specific activation of CASM, inducing GFP-hLC3A recruitment to endolysosomes, and a lipidation-associated band-shift (Figures 1A and 1B), with no significant effect on global lipid composition (Figures S1C and S1D). Strikingly, under these conditions, mass spectrometry detects GFP-hLC3A conjugated to both PE and PS (Figures 1C, 1D, 1G, and 1H). These data provide clear evidence for *in vivo*, cellular ATG8-PS conjugation. To broaden these findings, multiple ATG8 isoforms were tested (hLC3B/C; hGABARAP/L1/L2), and in each case, monensin drives alternative conjugation to PS (Figures S2A–S2G). Using normalized peak areas to estimate relative abundance, ATG8-PS represents approximately 10% (hLC3A) to 30% (hGABARAP) of the lipidated form, under these conditions. Similar results are also observed at endogenous expression levels (hGABARAPL2 knockin model, Figures 1I–1K) (Eck et al., 2020).

Collectively, these data establish that ATG8-PS lipidation can occur in cells, across all ATG8 isoforms, and indicate that non-canonical autophagy/CASM drives this distinctive modification.

ATG8-PS lipidation during physiological non-canonical autophagy

To extend these findings to more physiological processes, CASM was analyzed during LAP. Using J774A.1 macrophage, GFP-hLC3A recruitment to phagosomes housing IgG-coated beads was analyzed, in the presence or absence of BafA1 (Figures 2A and 2B); BafA1 inhibits CASM, a V-ATPase-dependent process (Florey et al., 2015; Gao et al., 2016), in contrast to its effects on canonical autophagy. As expected, BafA1 does not influence the number of phagosomes formed (Figure 2C) but does reduce levels of lipidated GFP-hLC3A-II during LAP (Figure 2D). These data also verify that the majority of enriched GFP-hLC3A derives from phagosomes, not contaminating autophagosomes (where BafA1 would instead increase GFP-LC3-II by blocking autophagosome flux). Importantly, induction of LAP drives the alternative lipidation of hLC3A with PS, as well as PE (Figures 2E and 2F). In this case, hLC3A-PS accounts for ~25% of the lipidated species and is reduced by BafA1 even more robustly than hLC3A-PE.

To investigate an additional physiological trigger, influenza A virus (IAV) infection was assessed in HCT116 cells, in which the viral M2 proton channel drives CASM (Fletcher et al., 2018), as shown by GFP-rLC3B lipidation (Figure 2G). Importantly, mass spectrometric analysis detects GFP-rLC3B conjugation to both PS and PE, with rLC3B-PS representing ~20% of the total lipidated species (Figures 2H and 2I).

Together, these data establish that ATG8-PS lipidation occurs broadly upon induction of CASM via pharmacological activation, LAP, or IAV infection.

Molecular mechanisms of ATG8-PS lipidation

To define the molecular mechanisms underpinning differential ATG8 lipidation, the contribution of ATG16L1 was examined. ATG16L1 is a molecular hub, coordinating autophagy pathways, via distinct domains, that support either canonical or non-canonical signaling (Dooley et al., 2014; Fletcher et al., 2018; Gammoh et al., 2013; Lystad et al., 2019; Rai et al., 2019). The ATG16L1 WD40 domain bears key residues which, when mutated (e.g., K490A), render cells competent for canonical autophagy but deficient for CASM (Fletcher et al., 2018; Lystad et al., 2019) and can be used to dissect these pathways. A panel of

(C) C-terminal peptides of hLC3A conjugated to the PE or PS headgroup. Predicted molecular weights (MWs) are indicated.

(D) Collision-induced dissociation (CID) mass spectra of unmodified, PE-modified, or PS-modified hLC3A C-terminal peptides. Monoisotopic mass shifts: 197.05, glycerophosphoethanolamine (from PE); 241.04, glycerophosphoserine (from PS); arrowheads denote y8 ion peaks as examples.

(E–H) Normalized mass spectrometry analysis of hLC3A-PE and hLC3A-PS in WT (E and F) and *ATG13*$^{-/-}$ (G and H) cells.

(I–K) Analysis of endogenous GABARAPL2 in HeLa cells by western blotting and mass spectrometry.

Data represent means from three independent experiments. *p < 0.03 and **p < 0.002, paired t test. See also Figure S2.

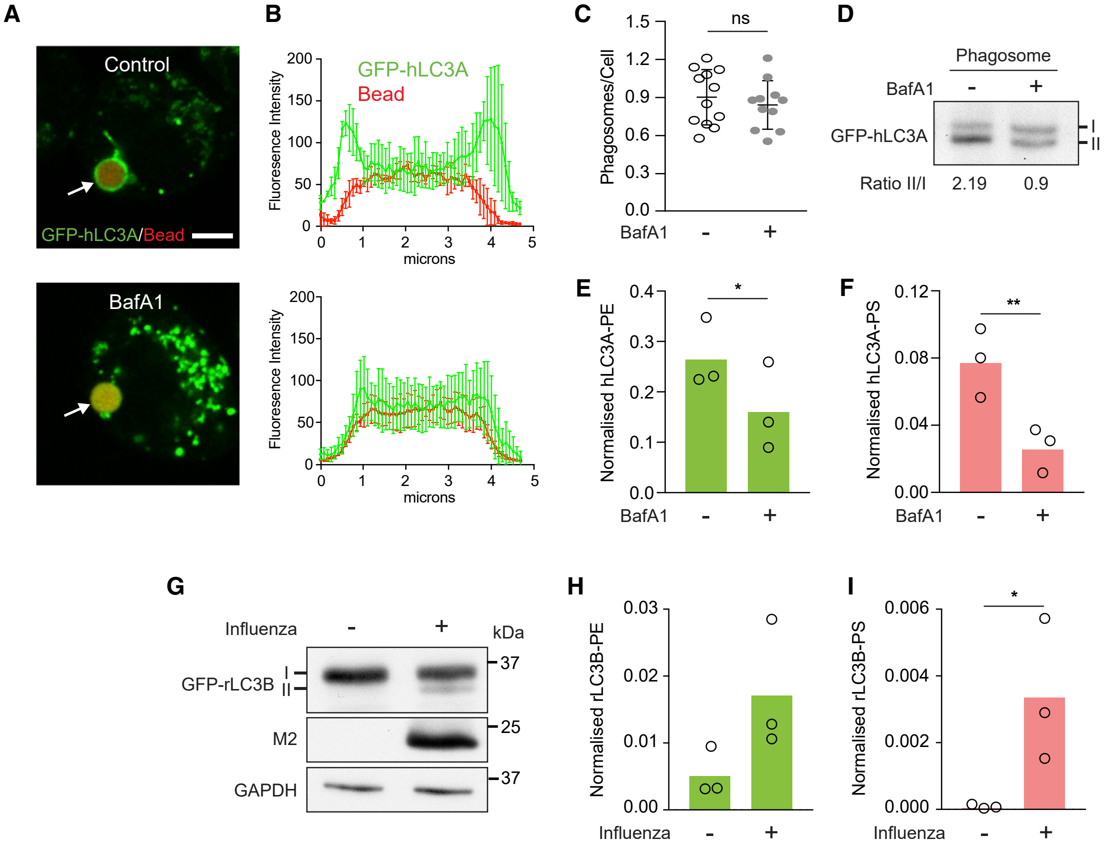

Figure 2. ATG8-PS lipidation occurs during LC3-associated phagocytosis (LAP) and influenza A virus (IAV) infection

(A) Confocal images of J774A.1 macrophage treated with IgG-coated beads to induce LAP −/+ BafA1. Scale bar: 5 μm.

(B) Signal intensity profile of phagosomal GFP-hLC3A. Data represent mean ± SD from three phagosomes.

(C) Quantification of phagocytosis. Data represent means ± SD from more than ten fields of view.

(D) Western blot of GFP-hLC3A from phagosome fraction with ratio of LC3II/LC3I.

(E and F) Normalized mass spectrometry analysis of hLC3A-PE and hLC3A-PS from phagosome fractions. Data represent means from three independent experiments. $^*p < 0.03$ and $^{**}p < 0.002$, paired t test.

(G) HCT116 cells infected with influenza A virus (IAV) PR8 and analyzed using western blot.

(H and I) Normalized mass spectrometry analysis of rLC3B-PE and rLC3B-PS −/+ IAV infection. Data represent means from three independent experiments. $^*p < 0.03$, ratio paired t test.

ATG16L1$^{-/-}$ HCT116 cells, reconstituted with either WT ATG16L1 or the K490A mutant, were thus analyzed. As expected, activation of canonical autophagy (PP242/BafA1) induces autophagosome formation (Figure 3A) and conjugation of GFP-rLC3B to PE, but not PS (Figures 3B and 3C), in WT and K490A cells equally (but not *ATG16L1*$^{-/-}$ controls). In contrast, induction of CASM yields differential results. In WT cells, monensin drives GFP-rLC3B relocalization to endolysosomes and lipidation to both PE and PS (Figures 3D–3G). However, K490A cells are completely deficient in rLC3B-PS lipidation; monensin does induce a small but reproducible increase in rLC3B-PE in these cells, likely through a block of basal autophagy flux (Figure 3F). These data show that ATG8-PS conjugation is dependent on the ATG16L1 WD40 domain. To extend these findings, ATG16L1 was assessed in RAW267.4 macrophage undergoing LAP (Figures S3A–S3C). Again, hLC3A-PS is detected in WT, but not K490A, cells. Together, these data demonstrate that ATG8-PS lipidation is completely dependent on the molecular machinery of non-canonical autophagy.

ATG16L1, in complex with ATG5/12, directs the site of ATG8 lipidation (Fujita et al., 2008). We thus reasoned that alternative ATG8 lipidation may result, at least in part, from differences in lipid composition at the distinct membranes targeted by ATG16L1 during CASM versus autophagy (Hanada et al., 2007). To investigate this, a fluorescent sensor for PS (Lact-C2) (Yeung et al., 2008) was expressed in cells undergoing different autophagy-related processes. PS is clearly enriched at various hLC3A-positive compartments during CASM, including phagosomes (Figure 3H), lysosomes, macropinosomes, and entotic vacuoles (Figures S3D–S3F). In contrast, PS could not be detected on forming autophagosomes (Figure 3I). These data support a simple model in which local PS availability may influence the identity of ATG8 lipidation, although other regulatory mechanisms may also operate.

CellPress
OPEN ACCESS

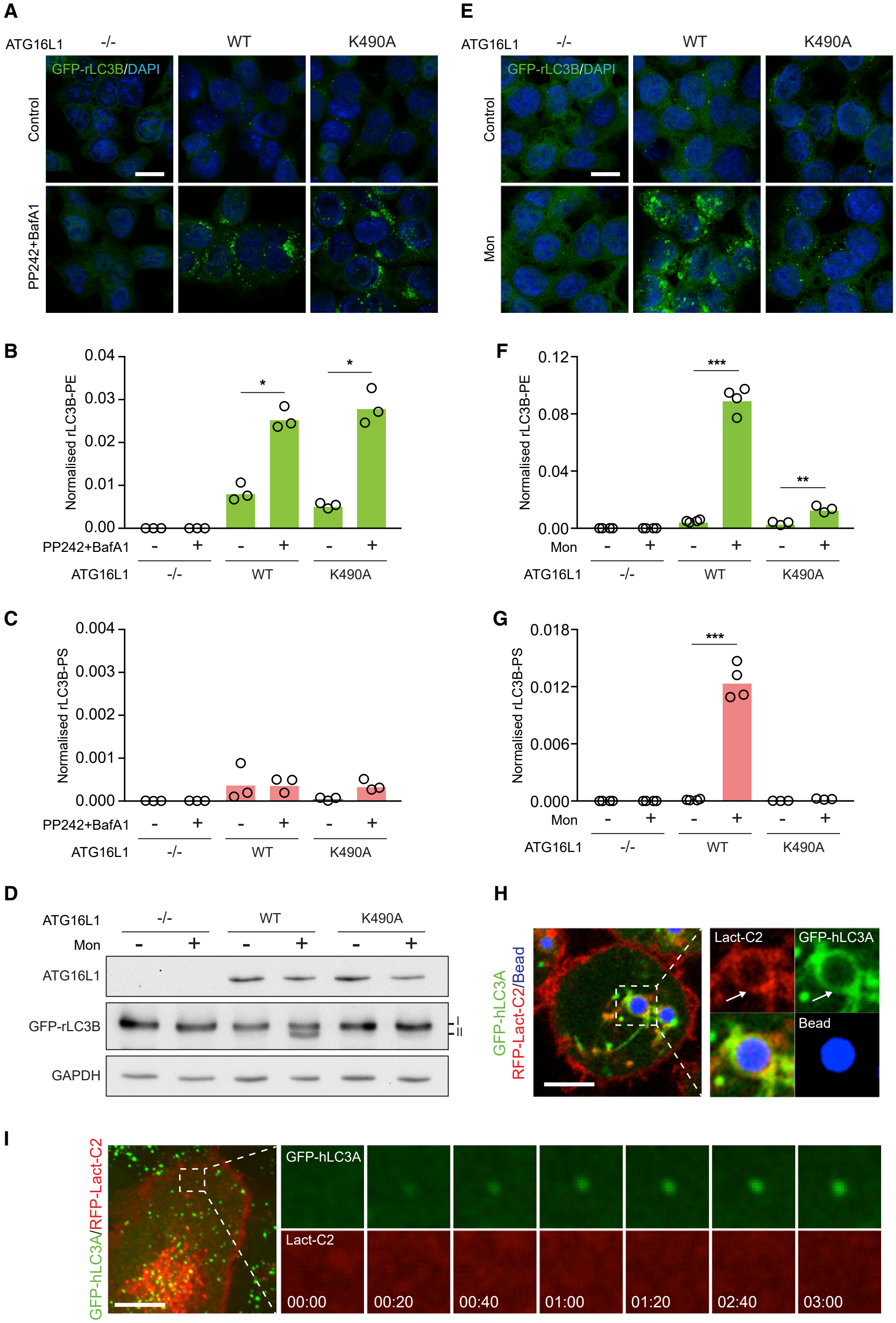

(legend on next page)

Collectively, these data indicate that the molecular machinery of non-canonical autophagy, such as the ATG16L1 WD40 domain, directs ATG8-PS conjugation at PS-enriched, endolysosomal single membranes.

Differential delipidation of ATG8-PS and PE by ATG4s

We next considered the molecular consequences of differential ATG8 conjugation, with a focus on ATG4s, the dual-activity proteases that prime pro-ATG8s and then catalyze subsequent delipidation. To explore this, conjugation of PE or PS was modeled onto the LC3B(120)-ATG4B co-complex structure (Figure 4A) (Satoo et al., 2009). These phospholipids differ by just a single carboxyl group, which confers extra bulk and negative charge to PS. Notably, modeling suggests this distinctive moiety would juxtapose with ATG4B Trp142, a residue critical for structure and activity (Sugawara et al., 2005). As such, the additional PS carboxyl group may limit freedom of movement and sterically hinder delipidation. To test this, a mixed pool of CASM-induced hLC3A-PS and hLC3A-PE was enriched from cells and incubated with recombinant ATG4B *in vitro* (Figure 4B). Strikingly, whereas hLC3A-PE undergoes robust delipidation through time (Figure S4A), and across experiments (Figure 4C), hLC3A-PS is largely resistant to deconjugation under these conditions. These data confirm that differential ATG8 lipidation can influence ATG4B-mediated deconjugation, revealing a functional outcome for this alternative modification. These findings are consistent with previous *in vitro* analyses of GABARAPL1 liposomes, delipidated by ATG4A, B, or C (Kauffman et al., 2018), suggesting that a reduced rate of ATG8-PS cleavage may be shared among multiple isoforms of both ATG8 and ATG4.

However, CASM is a transient and reversible process (Florey et al., 2011), implying that delipidation of both species is likely to occur in cells. As such, we reasoned that an alternative ATG4 isoform may catalyze PS deconjugation. To investigate this, ATG4 proteins (A–D) were purified from mammalian cells, and their delipidation profiles assayed, using ATG8 substrates (hLC3B or hGABARAP) conjugated to liposomes (PE or PS) (Figures 4D–4F). Notably, ATG4A is GABARAP specific, and full-length ATG4C/D are active under these conditions, unlike the bacterially purified proteins (Betin and Lane, 2009; Kauffman et al., 2018). RavZ, a bacterial effector protein known to cleave both ATG8-PE and PS (Choy et al., 2012; Yang et al., 2017), was included as a positive control. Consistent with published work, all four ATG4s can delipidate ATG8s from PE liposomes, to varying degrees (Kauffman et al., 2018). However, ATG4D preferentially deconjugates both hLC3B-PS and hGABARAP-PS, uncovering a specific function for this isoform. Notably, ATG4B also supports partial ATG8-PS deconjugation on liposomes, suggesting that altered conditions, such as membrane curvature and/or charge, may enable this activity.

To control for any indirect effects of liposome composition, a mixed lipid system was assessed, in which hLC3B is conjugated to PE or PS, on the same liposomes, and delipidation is measured by mass spectrometry (Figure 4G). Here too, hLC3B-PS is more efficiently delipidated by ATG4D than ATG4B, while hLC3B-PE is deconjugated well by both. Together, these data indicate that ATG4s display isoform specificity, with differential activities toward ATG8-PE and ATG8-PS substrates.

To develop these findings, cellular ATG4 activity was investigated using CRISPR deletion. Consistent with *in vitro* observations, loss of *ATG4D* (HCT116 cells) elevates cellular levels of rLC3B-PS and rLC3B-PE, during monensin-induced CASM (Figures 4H and S4B). These data reinforce the notion that ATG4D provides a major PS-delipidating activity in the cell. We also tested ATG4B, which can mediate ATG8-PS deconjugation *in vitro*, although this reaction is structurally disfavored and relatively inefficient. Given that ATG4B is essential for the activation of pro-ATG8, pre-primed GFP-hLC3B (G120) was expressed, in WT or $ATG4B^{-/-}$ HeLa cells (Figures 4I and S4C) (Agrotis et al., 2019). Interestingly, but somewhat surprisingly, loss of cellular ATG4B elevates levels of hLC3B-PS (and hLC3B-PE) during CASM, in a similar manner to ATG4D deletion. These data suggest that under cellular conditions, both ATG4 isoforms can support ATG8-PS deconjugation.

Finally, the overall cellular dynamics of ATG8-PS and ATG8-PE were compared during CASM. LAP was induced in RAW264.7 cells (Figures 4J and 4K), and lipid conjugation quantified over time. As expected, LAP drives the conjugation of hLC3A to both PS and PE, increasing over time, then falling again (Figures S4D and S4E). Notably, ratiometric analysis shows the two species bear different kinetics, with hLC3A-PS persisting for longer (Figure 4L). These data indicate that the balance of ATG4-delipidating activities favors the more rapid processing of ATG8-PE, with ATG8-PS representing a longer lived species. Collectively, these findings establish clear functional differences between ATG8-PS and ATG8-PE with respect to ATG4 deconjugation and associated signaling dynamics.

DISCUSSION

The C-terminal lipidation of ATG8 is a unique post-translational modification and a hallmark event during autophagy-related processes, widely used to detect and monitor the pathway. Here, we provide evidence for alternative ATG8 lipidation to PS, during non-canonical autophagy, thereby bridging the seminal studies

Figure 3. The ATG16L1 WD40 domain supports alternative ATG8 lipidation, which occurs at PS-enriched membranes
(A) Confocal images of HCT116 $ATG16L1^{-/-}$ cells, re-expressing ATG16L1 WT or K490A, stimulated for canonical autophagy (PP242/BafA1). Scale bar: 20 μm.
(B and C) Normalized mass spectrometry analysis of rLC3B-PE and rLC3B-PS in cells treated as in (A).
(D) Western blot analysis of HCT116 cell panel, stimulated −/+ monensin.
(E) Confocal images of HCT116 cells treated as in (D).
(F and G) Normalized mass spectrometry analysis of rLC3B-PE and rLC3B-PS in cells treated as in (D).
(H) Confocal images of GFP-hLC3A and RFP-Lact-C2 in J774.A1 cells during LAP. Scale bar: 5 μm; arrows denote a GFP-hLC3A-positive phagosome.
(I) Live confocal imaging of MCF10A cells, expressing GFP-hLC3A and RFP-Lact-C2, treated with PP242. Scale bar: 5 μm. Cropped time-lapse frames, min:sec.
Data represent means from three or four independent experiments. *p < 0.03, **p < 0.002, and ***p < 0.0002, paired t test. See also Figure S3.

CellPress
OPEN ACCESS

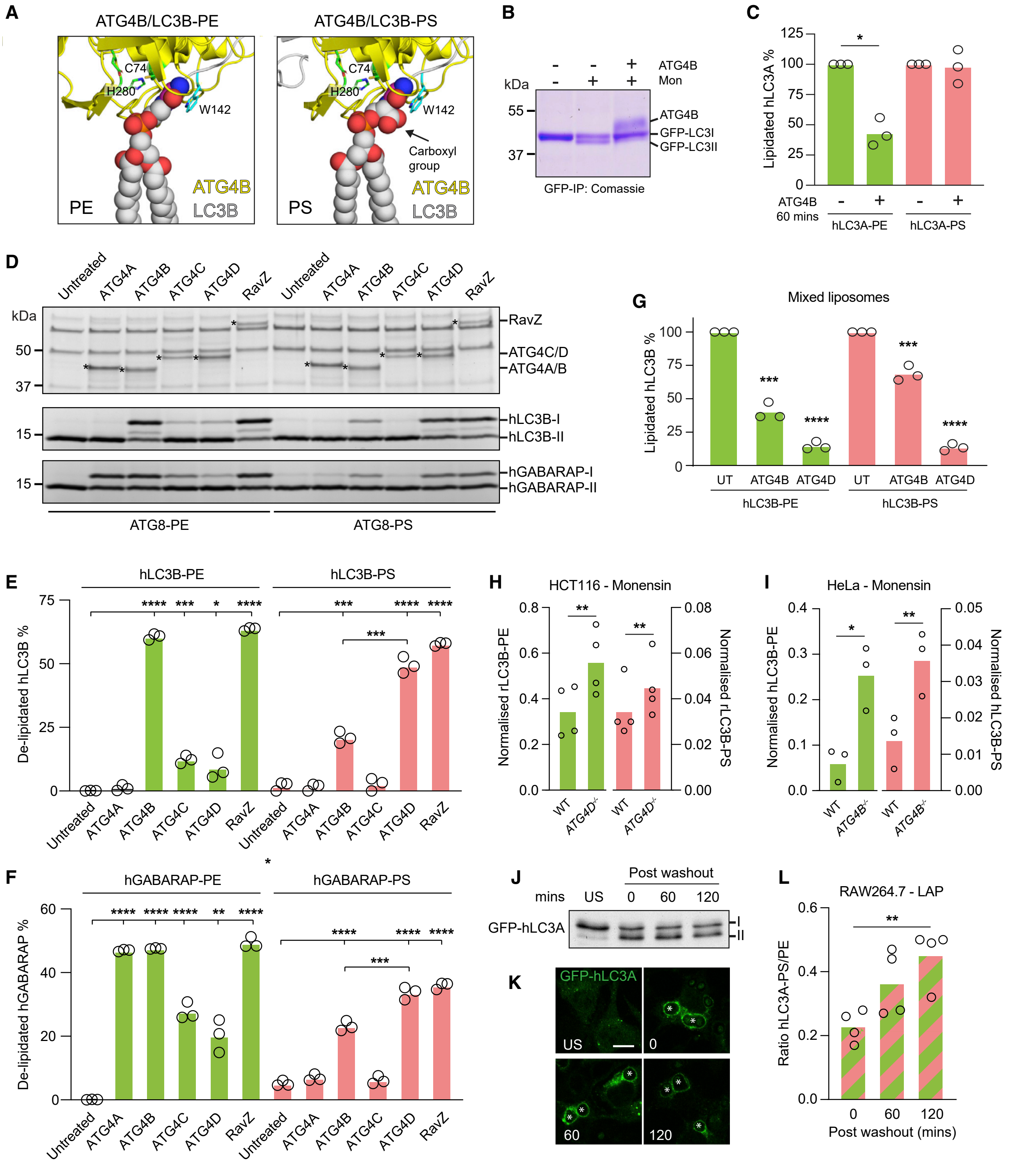

Figure 4. ATG8-PS and ATG8-PE undergo differential delipidation by the ATG4 family

(A) Molecular modeling of LC3B-PE and LC3B-PS in complex with ATG4B (on the basis of PDB: 2Z0D), with critical catalytic residues marked.

(B) Coomassie staining of GFP-hLC3A IPs from MCF10A *ATG13*$^{-/-}$ cells −/+ monensin, incubated −/+ ATG4B for 60 min.

(C) Mass spectrometry analysis of hLC3A-PE and hLC3A-PS from cells treated as in (B). Data represent three independent experiments with means normalized to time 0. *$p < 0.01$, paired t test.

(D) PE or PS liposome-based delipidation assays with purified ATG4s or RavZ. Conjugated hLC3B or hGABARAP was incubated with ATG4A/B/C/D/RavZ (asterisk) for 60 min and analyzed using SDS-PAGE/Coomassie.

(legend continued on next page)

of ATG8 lipid conjugation to more recent insights into the broader autophagy landscape.

Alternative ATG8 lipidation occurs during pharmacological CASM, LAP and influenza A infection, at single-membrane endolysosomal compartments, enriched in PS. In a striking dichotomy, ATG8-PS is not detected during canonical autophagy, consistent with published work (Ichimura et al., 2000). As such, ATG8-PS may provide a "molecular signature" for non-canonical autophagy, enabling its distinction from closely related, parallel pathways. It will be interesting to determine whether ATG8-PS is detected in other physiological contexts.

ATG8-PS and ATG8-PE are differentially deconjugated by ATG4 isoforms and exhibit altered signaling dynamics in cells, revealing clear molecular distinctions between these species. ATG4 isoform specificity has been well studied with respect to proteolytic priming and PE delipidation (Kauffman et al., 2018), and our findings build further on these insights. Although ATG4 proteolytic activity is quite promiscuous, with many amino acids accommodated downstream of the scissile Gly (Sugawara et al., 2005), delipidating activity appears more selective, likely because of the structural constraints of the lipid headgroup. Our data indicate that ATG4D, and ATG4B, can catalyze ATG8-PS delipidation during CASM.

ATG4D has not been comprehensively studied, although previous work has identified links to mitochondria and apoptosis (Betin and Lane, 2009). Our data support a key role for ATG4D during non-canonical autophagy. Consistent with this, ATG4D was identified as a modulator of LC3 lipidation during IAV infection (Ulferts et al., 2020). It will be interesting to establish whether this activity affects viral responses (Wang et al., 2020) and/or the neuronal phenotypes observed in ATG4D deficient models (Kyöstilä et al., 2015; Syrjä et al., 2017).

ATG4B also catalyzes ATG8-PS deconjugation in cells, though this activity is structurally unfavorable and inefficient *in vitro*. It seems likely that cellular mechanisms can modulate ATG4B selectivity. For instance, ATG4B modifications have been reported, and it would be interesting to assess whether these influence activity (Pengo et al., 2017). Further analyses of ATG4 enzyme kinetics, expression, localization, post-translational modification, and knockout (KO) phenotypes will be required to define exactly how their activities differ, and can be regulated, during CASM and other autophagy contexts.

Differentially lipidated ATG8s bear altered dynamics, with ATG8-PS persisting longer during LAP. Future work will interrogate the functional role(s) of this species more comprehensively. It is tempting to speculate that conjugation of PS to ATG8 may enable binding to distinct interacting partners, to couple to alternative signaling pathways. Conversely, it is possible that ATG8 conjugation might instead influence the properties of PS, which mediates critical charge effects during phagocytosis (Yeung et al., 2009).

Collectively, our findings open up a range of important mechanistic and functional questions related to ATG8s and ATG4s, in different autophagy contexts, to explore through future study.

Limitations

This study identifies and characterizes cellular ATG8 conjugation to PS and its impact on ATG4s. It will be interesting next to investigate the physiological functions of this unique modification. This will depend upon the development of tools to specifically promote or inhibit conjugation to PS, rather than PE, which are not yet available but will form the focus of future work.

STAR★METHODS

Detailed methods are provided in the online version of this paper and include the following:

- KEY RESOURCES TABLE
- RESOURCE AVAILABILITY
  - Lead contact
  - Materials availability
  - Data and code availability
- EXPERIMENTAL MODEL AND SUBJECT DETAILS
- METHOD DETAILS
  - Reagents
  - Plasmids
  - Generation of ATG4D CRISPR knock out cells
  - Retrovirus production and infection
  - Pharmacological stimulation
  - J774.A1 phagosome preparation and assay
  - RAW264.7 phagocytosis assay
  - Influenza A infection
  - Whole cell lipidomic analysis
  - Cell lysis and GFP-TRAP immunoprecipitation
  - HA-immunoprecipitation
  - Mass spectrometric analysis of lipidated ATG8
  - On bead ATG4B delipidation assay
  - Protein purification for liposome assays
  - Liposome assays
  - Western blotting

(E and F) Densitometry analysis of (D). Data represent means from three independent experiments *$p < 0.03$, **$p < 0.002$, ***$p < 0.0002$, and ****$p < 0.0001$, unpaired t test.
(G) Mass spectrometry analysis of hLC3B conjugation on mixed liposomes, incubated with ATG4B or ATG4D for 60 min. Data represent means normalized to untreated controls from three independent experiments. ***$p < 0.0002$ and ****$p < 0.0001$, unpaired t test.
(H and I) Normalized mass spectrometry analysis of GFP-rLC3B from monensin treated WT and *ATG4D*$^{-/-}$ HCT116 cells (H) and of GFP-hLC3BG120 from monensin treated WT and *ATG4B*$^{-/-}$ HeLa cells (I). Data represent means from three or four independent experiments. *$p < 0.03$ and **$p < 0.002$, paired t test.
(J) Western blot analysis of RAW264.7 cells expressing GFP-hLC3A treated −/+ zymosan for 25 min, followed by washout 0–120 min post-LAP.
(K) Confocal images of cells treated as in (J). Scale bar: 5 μm. Asterisks denote phagosomes.
(L) Ratios of hLC3A-PS/PE measured by mass spectrometry from cells treated as in (J). Data represent means from four independent experiments. **$p < 0.002$, unpaired t test.
See also Figure S4.

- ○ Microscopy
- ○ LC3B-ATG4 complex modeling
- ● QUANTIFICATION AND STATISTICAL ANALYSIS

SUPPLEMENTAL INFORMATION

Supplemental information can be found online at https://doi.org/10.1016/j.molcel.2021.03.020.

ACKNOWLEDGMENTS

We are grateful to Kranthikumar Yadav G for technical support. We thank Nick Ktistakis, Len Stephens, Phill Hawkins, Simon Cook, and members of the Florey lab for helpful discussions and critical review of the manuscript. This work was supported by grants from the Biotechnology and Biological Sciences Research Council (BBSRC), BB/P013384/1 (BBS/E/B/000C0432 and BBS/E/B/000C0434), BB/R019258/1, and Cancer Research UK Career Development Award C47718/A16337. The Babraham Institute (BI) Mass Spectrometry Facility was supported by a BBSRC Core Capability Grant. This work was partly supported by the Research Council of Norway, through its Centres of Excellence funding scheme (project 262652). We dedicate this work to our friend and colleague Prof. Michael Wakelam, who sadly passed away in March 2020.

AUTHOR CONTRIBUTIONS

J.D. and O.F. conceived of, designed, and carried out experiments and wrote the paper. A.H.L. designed and performed liposome assays. K.S. generated cell lines and carried out experiments (GABARAP and RAW264.7 cell LAP assays). S.R.C. purified ATG4 and RavZ proteins. M.I.W. generated computational models of ATG4B-LC3-II. E.M. and R.U. characterized the ATG4D CRISPR KO cell line. A.F.L.-C. and M.J.W. analyzed global lipidomics. D.O. and J.W. designed the mass spectrometry experiments and generated data. A.S. and R.B. provided reagents and expertise.

DECLARATION OF INTERESTS

The authors declare no competing interests.

Received: July 24, 2020
Revised: January 15, 2021
Accepted: March 16, 2021
Published: April 27, 2021

REFERENCES

Acheson, J.F., Derewenda, Z.S., and Zimmer, J. (2019). Architecture of the cellulose synthase outer membrane channel and its association with the periplasmic TPR domain. Structure *27*, 1855–1861.e3.

Agrotis, A., Pengo, N., Burden, J.J., and Ketteler, R. (2019). Redundancy of human ATG4 protease isoforms in autophagy and LC3/GABARAP processing revealed in cells. Autophagy *15*, 976–997.

Betin, V.M., and Lane, J.D. (2009). Caspase cleavage of Atg4D stimulates GABARAP-L1 processing and triggers mitochondrial targeting and apoptosis. J. Cell Sci. *122*, 2554–2566.

Choy, A., Dancourt, J., Mugo, B., O'Connor, T.J., Isberg, R.R., Melia, T.J., and Roy, C.R. (2012). The Legionella effector RavZ inhibits host autophagy through irreversible Atg8 deconjugation. Science *338*, 1072–1076.

Cunha, L.D., Yang, M., Carter, R., Guy, C., Harris, L., Crawford, J.C., Quarato, G., Boada-Romero, E., Kalkavan, H., Johnson, M.D.L., et al. (2018). LC3-associated phagocytosis in myeloid cells promotes tumor immune tolerance. Cell *175*, 429–441.e16.

de Wit, E., Spronken, M.I., Bestebroer, T.M., Rimmelzwaan, G.F., Osterhaus, A.D., and Fouchier, R.A. (2004). Efficient generation and growth of influenza virus A/PR/8/34 from eight cDNA fragments. Virus Res. *103*, 155–161.

Dooley, H.C., Razi, M., Polson, H.E., Girardin, S.E., Wilson, M.I., and Tooze, S.A. (2014). WIPI2 links LC3 conjugation with PI3P, autophagosome formation, and pathogen clearance by recruiting Atg12-5-16L1. Mol. Cell *55*, 238–252.

Durgan, J., Tseng, Y.Y., Hamann, J.C., Domart, M.C., Collinson, L., Hall, A., Overholtzer, M., and Florey, O. (2017). Mitosis can drive cell cannibalism through entosis. eLife *6*, e27134.

Eck, F., Phuyal, S., Smith, M.D., Kaulich, M., Wilkinson, S., Farhan, H., and Behrends, C. (2020). ACSL3 is a novel GABARAPL2 interactor that links ufmylation and lipid droplet biogenesis. J. Cell Sci. *133*, jcs243477.

Fischer, T.D., Wang, C., Padman, B.S., Lazarou, M., and Youle, R.J. (2020). STING induces LC3B lipidation onto single-membrane vesicles via the V-ATPase and ATG16L1-WD40 domain. J. Cell Biol. *219*, e202009128.

Fletcher, K., Ulferts, R., Jacquin, E., Veith, T., Gammoh, N., Arasteh, J.M., Mayer, U., Carding, S.R., Wileman, T., Beale, R., and Florey, O. (2018). The WD40 domain of ATG16L1 is required for its non-canonical role in lipidation of LC3 at single membranes. EMBO J. *37*, e97840.

Florey, O., Kim, S.E., Sandoval, C.P., Haynes, C.M., and Overholtzer, M. (2011). Autophagy machinery mediates macroendocytic processing and entotic cell death by targeting single membranes. Nat. Cell Biol. *13*, 1335–1343.

Florey, O., Gammoh, N., Kim, S.E., Jiang, X., and Overholtzer, M. (2015). V-ATPase and osmotic imbalances activate endolysosomal LC3 lipidation. Autophagy *11*, 88–99.

Fujita, N., Itoh, T., Omori, H., Fukuda, M., Noda, T., and Yoshimori, T. (2008). The Atg16L complex specifies the site of LC3 lipidation for membrane biogenesis in autophagy. Mol. Biol. Cell *19*, 2092–2100.

Galluzzi, L., and Green, D.R. (2019). Autophagy-independent functions of the autophagy machinery. Cell *177*, 1682–1699.

Gammoh, N., Florey, O., Overholtzer, M., and Jiang, X. (2013). Interaction between FIP200 and ATG16L1 distinguishes ULK1 complex-dependent and -independent autophagy. Nat. Struct. Mol. Biol. *20*, 144–149.

Gao, Y., Liu, Y., Hong, L., Yang, Z., Cai, X., Chen, X., Fu, Y., Lin, Y., Wen, W., Li, S., et al. (2016). Golgi-associated LC3 lipidation requires V-ATPase in noncanonical autophagy. Cell Death Dis. *7*, e2330.

Hanada, T., Noda, N.N., Satomi, Y., Ichimura, Y., Fujioka, Y., Takao, T., Inagaki, F., and Ohsumi, Y. (2007). The Atg12-Atg5 conjugate has a novel E3-like activity for protein lipidation in autophagy. J. Biol. Chem. *282*, 37298–37302.

Hartler, J., Triebl, A., Ziegl, A., Trötzmüller, M., Rechberger, G.N., Zeleznik, O.A., Zierler, K.A., Torta, F., Cazenave-Gassiot, A., Wenk, M.R., et al. (2017). Deciphering lipid structures based on platform-independent decision rules. Nat. Methods *14*, 1171–1174.

Heckmann, B.L., Teubner, B.J.W., Tummers, B., Boada-Romero, E., Harris, L., Yang, M., Guy, C.S., Zakharenko, S.S., and Green, D.R. (2019). LC3-associated endocytosis facilitates β-amyloid clearance and mitigates neurodegeneration in murine Alzheimer's disease. Cell *178*, 536–551.e14.

Henault, J., Martinez, J., Riggs, J.M., Tian, J., Mehta, P., Clarke, L., Sasai, M., Latz, E., Brinkmann, M.M., Iwasaki, A., et al. (2012). Noncanonical autophagy is required for type I interferon secretion in response to DNA-immune complexes. Immunity *37*, 986–997.

Ichimura, Y., Kirisako, T., Takao, T., Satomi, Y., Shimonishi, Y., Ishihara, N., Mizushima, N., Tanida, I., Kominami, E., Ohsumi, M., et al. (2000). A ubiquitin-like system mediates protein lipidation. Nature *408*, 488–492.

Jacquin, E., Leclerc-Mercier, S., Judon, C., Blanchard, E., Fraitag, S., and Florey, O. (2017). Pharmacological modulators of autophagy activate a parallel noncanonical pathway driving unconventional LC3 lipidation. Autophagy *13*, 854–867.

Jacquin, E., Fletcher, K., and Florey, O. (2019). Imaging noncanonical autophagy and LC3-associated phagocytosis in cultured cells. Methods Mol. Biol. *1880*, 295–303.

Johansen, T., and Lamark, T. (2020). Selective autophagy: ATG8 family proteins, LIR motifs and cargo receptors. J. Mol. Biol. *432*, 80–103.

Kauffman, K.J., Yu, S., Jin, J., Mugo, B., Nguyen, N., O'Brien, A., Nag, S., Lystad, A.H., and Melia, T.J. (2018). Delipidation of mammalian Atg8-family proteins by each of the four ATG4 proteases. Autophagy *14*, 992–1010.

Kim, J.Y., Zhao, H., Martinez, J., Doggett, T.A., Kolesnikov, A.V., Tang, P.H., Ablonczy, Z., Chan, C.C., Zhou, Z., Green, D.R., and Ferguson, T.A. (2013). Noncanonical autophagy promotes the visual cycle. Cell *154*, 365–376.

Kirisako, T., Ichimura, Y., Okada, H., Kabeya, Y., Mizushima, N., Yoshimori, T., Ohsumi, M., Takao, T., Noda, T., and Ohsumi, Y. (2000). The reversible modification regulates the membrane-binding state of Apg8/Aut7 essential for autophagy and the cytoplasm to vacuole targeting pathway. J. Cell Biol. *151*, 263–276.

Klionsky, D.J., Abdelmohsen, K., Abe, A., Abedin, M.J., Abeliovich, H., Acevedo Arozena, A., Adachi, H., Adams, C.M., Adams, P.D., Adeli, K., et al. (2016). Guidelines for the use and interpretation of assays for monitoring autophagy (3rd edition). Autophagy *12*, 1–222.

Kyöstilä, K., Syrjä, P., Jagannathan, V., Chandrasekar, G., Jokinen, T.S., Seppälä, E.H., Becker, D., Drögemüller, M., Dietschi, E., Drögemüller, C., et al. (2015). A missense change in the ATG4D gene links aberrant autophagy to a neurodegenerative vacuolar storage disease. PLoS Genet. *11*, e1005169.

Lystad, A.H., Carlsson, S.R., de la Ballina, L.R., Kauffman, K.J., Nag, S., Yoshimori, T., Melia, T.J., and Simonsen, A. (2019). Distinct functions of ATG16L1 isoforms in membrane binding and LC3B lipidation in autophagy-related processes. Nat. Cell Biol. *21*, 372–383.

Ma, J., Becker, C., Lowell, C.A., and Underhill, D.M. (2012). Dectin-1-triggered recruitment of light chain 3 protein to phagosomes facilitates major histocompatibility complex class II presentation of fungal-derived antigens. J. Biol. Chem. *287*, 34149–34156.

Martinez, J., Malireddi, R.K., Lu, Q., Cunha, L.D., Pelletier, S., Gingras, S., Orchard, R., Guan, J.L., Tan, H., Peng, J., et al. (2015). Molecular characterization of LC3-associated phagocytosis reveals distinct roles for Rubicon, NOX2 and autophagy proteins. Nat. Cell Biol. *17*, 893–906.

Martinez, J., Cunha, L.D., Park, S., Yang, M., Lu, Q., Orchard, R., Li, Q.Z., Yan, M., Janke, L., Guy, C., et al. (2016). Noncanonical autophagy inhibits the autoinflammatory, lupus-like response to dying cells. Nature *533*, 115–119.

Mizushima, N. (2020). The ATG conjugation systems in autophagy. Curr. Opin. Cell Biol. *63*, 1–10.

Mizushima, N., and Yoshimori, T. (2007). How to interpret LC3 immunoblotting. Autophagy *3*, 542–545.

Nakatogawa, H., Oh-oka, K., and Ohsumi, Y. (2008). Lipidation of Atg8: how is substrate specificity determined without a canonical E3 enzyme? Autophagy *4*, 911–913.

Nguyen, T.N., Padman, B.S., Usher, J., Oorschot, V., Ramm, G., and Lazarou, M. (2016). Atg8 family LC3/GABARAP proteins are crucial for autophagosome-lysosome fusion but not autophagosome formation during PINK1/Parkin mitophagy and starvation. J. Cell Biol. *215*, 857–874.

Oh-oka, K., Nakatogawa, H., and Ohsumi, Y. (2008). Physiological pH and acidic phospholipids contribute to substrate specificity in lipidation of Atg8. J. Biol. Chem. *283*, 21847–21852.

Pengo, N., Agrotis, A., Prak, K., Jones, J., and Ketteler, R. (2017). A reversible phospho-switch mediated by ULK1 regulates the activity of autophagy protease ATG4B. Nat. Commun. *8*, 294.

Rai, S., Arasteh, M., Jefferson, M., Pearson, T., Wang, Y., Zhang, W., Bicsak, B., Divekar, D., Powell, P.P., Naumann, R., et al. (2019). The ATG5-binding and coiled coil domains of ATG16L1 maintain autophagy and tissue homeostasis in mice independently of the WD domain required for LC3-associated phagocytosis. Autophagy *15*, 599–612.

Sanjuan, M.A., Dillon, C.P., Tait, S.W., Moshiach, S., Dorsey, F., Connell, S., Komatsu, M., Tanaka, K., Cleveland, J.L., Withoff, S., and Green, D.R. (2007). Toll-like receptor signalling in macrophages links the autophagy pathway to phagocytosis. Nature *450*, 1253–1257.

Santiago, C., Ballesteros, A., Martínez-Muñoz, L., Mellado, M., Kaplan, G.G., Freeman, G.J., and Casasnovas, J.M. (2007). Structures of T cell immunoglobulin mucin protein 4 show a metal-Ion-dependent ligand binding site where phosphatidylserine binds. Immunity *27*, 941–951.

Satoo, K., Noda, N.N., Kumeta, H., Fujioka, Y., Mizushima, N., Ohsumi, Y., and Inagaki, F. (2009). The structure of Atg4B-LC3 complex reveals the mechanism of LC3 processing and delipidation during autophagy. EMBO J. *28*, 1341–1350.

Sou, Y.S., Tanida, I., Komatsu, M., Ueno, T., and Kominami, E. (2006). Phosphatidylserine in addition to phosphatidylethanolamine is an in vitro target of the mammalian Atg8 modifiers, LC3, GABARAP, and GATE-16. J. Biol. Chem. *281*, 3017–3024.

Sugawara, K., Suzuki, N.N., Fujioka, Y., Mizushima, N., Ohsumi, Y., and Inagaki, F. (2005). Structural basis for the specificity and catalysis of human Atg4B responsible for mammalian autophagy. J. Biol. Chem. *280*, 40058–40065.

Syrjä, P., Anwar, T., Jokinen, T., Kyöstilä, K., Jäderlund, K.H., Cozzi, F., Rohdin, C., Hahn, K., Wohlsein, P., Baumgärtner, W., et al. (2017). Basal autophagy is altered in Lagotto Romagnolo dogs with an ATG4D mutation. Vet. Pathol. *54*, 953–963.

Tanida, I., Ueno, T., and Kominami, E. (2004). Human light chain 3/MAP1LC3B is cleaved at its carboxyl-terminal Met121 to expose Gly120 for lipidation and targeting to autophagosomal membranes. J. Biol. Chem. *279*, 47704–47710.

Ulferts, R., Marcassa, E., Timimi, L., Lee, L.C., Daley, A., Montaner, B., Turner, S.D., Florey, O., Baillie, J.K., and Beale, R. (2020). Subtractive CRISPR screen identifies factors involved in non-canonical LC3 lipidation. bioRxiv, 2020.11.18.388306.

Wang, Y., Zhang, W., Jefferson, M., Sharma, P., Bone, B., Kipar, A., Coombes, J.L., Pearson, T., Man, A., Zhekova, A., et al. (2020). The WD and linker domains of ATG16L1 required for non-canonical autophagy limit lethal respiratory infection by influenza A virus at epithelial surfaces. bioRxiv, 2020.01.15.907873.

Yang, A., Pantoom, S., and Wu, Y.W. (2017). Elucidation of the anti-autophagy mechanism of the *Legionella* effector RavZ using semisynthetic LC3 proteins. eLife *6*, e23905.

Yeung, T., Gilbert, G.E., Shi, J., Silvius, J., Kapus, A., and Grinstein, S. (2008). Membrane phosphatidylserine regulates surface charge and protein localization. Science *319*, 210–213.

Yeung, T., Heit, B., Dubuisson, J.F., Fairn, G.D., Chiu, B., Inman, R., Kapus, A., Swanson, M., and Grinstein, S. (2009). Contribution of phosphatidylserine to membrane surface charge and protein targeting during phagosome maturation. J. Cell Biol. *185*, 917–928.

Zhuang, X., Magri, A., Hill, M., Lai, A.G., Kumar, A., Rambhatla, S.B., Donald, C.L., Lopez-Clavijo, A.F., Rudge, S., Pinnick, K., et al. (2019). The circadian clock components BMAL1 and REV-ERBα regulate flavivirus replication. Nat. Commun. *10*, 377.

CellPress
OPEN ACCESS

STAR★METHODS

KEY RESOURCES TABLE

REAGENT or RESOURCE	SOURCE	IDENTIFIER
**Antibodies**		
Rabbit Anti-ATG13 Monoclonal Antibody	Cell Signaling	Cat#13468; RRID: AB_2797419
Rabbit Anti-ATG16L1 Monoclonal Antibody	Cell Signaling	Cat#8089; RRID: AB_10950320
Anti-GAPDH Antibody	Abcam	Cat#ab9484; ab9484, RRID: AB_307274
Mouse Anti-GFP Monoclonal Antibody	Sigma	Cat#11814460001; RRID: AB_390913
Anti-M2 Antibody	Abcam	Cat#ab5416; RRID: AB_304873
Goat Anti-Rabbit IgG HRP Conjugated Antibody	Cell Signaling	Cat#7074; RRID: AB_2099233
Goat Anti-Mouse IgG HRP Conjugated Antibody	Cell Signaling	Cat#7076; RRID: AB_330924
Rabbit Anti-GABARAPL2 Polyclonal Antibody	In house	N/A
Rat Anti-HA Monoclonal Antibody	Roche	Cat#11867423001; RRID: AB_390918
Rabbit Anti-ATG4D Polyclonal Antibody	Proteintech	Cat#16924-1-AP; RRID: AB_2062024
Rabbit Anti-ATG4B Polyclonal Antibody	Cell Signaling	Cat#5299; RRID: AB_10622184
**Bacterial and virus strains**		
Influenza A Virus PR8 (strain A/Puerto Rico/9/1934)	Fletcher et al., 2018	N/A
BL21-Gold (DE3) *E. coli*.	Agilent	Cat#230132
**Chemicals, peptides, and recombinant proteins**		
Bafilomycin A1	Tocris	Cat#1334
PP242	Tocris	Cat#4257
Monensin	Sigma	Cat#M5273
DAPI	Sigma	Cat#D9542
Human IgG	Sigma	Cat#I4506
Murine IFNγ	Peprotech	Cat#315-05
GFP-TRAP beads	Chromotek	Cat#gtma-20
Control magnetic agarose beads	Chromotek	Cat#bmab-20
Magnetic 3-micron beads	Bangs Lanoratories	Cat#PMA3N
Latex 3-micron beads	Polysciences	Cat#17134-15
Zymosan	Sigma	Cat#Z4250
Human serum	Sigma	Cat#P2918
DMEM	Thermofisher	Cat#41966-029
DMEM F/12	Thermofisher	Cat#11320074
Pen/Strep	Thermofisher	Cat#15140-122
N-Ethylmaleimide (NEM)	Sigma	Cat#E3876
Puromycin	Sigma	Cat#P8833
Blasticidin	Sigma	Cat#15205
Protease inhibitor cocktail III	Sigma	Cat#P8340
Phosphatase inhibitor	Sigma	Cat#P0044
EGF	Peprotech	Cat#AF-100-15
Hydrocortisone	Sigma	Cat#H0888
Cholera toxin	Sigma	Cat#C8052
Insulin	Sigma	Cat#I9278
2x LDS buffer	Thermofisher	Cat#NP0008
Imperial Stain	Thermofisher	Cat#24615
AspN protease	Sigma	Cat#11420488001
Gold anti-fade	Thermofisher	Cat#P36930

(*Continued on next page*)

Continued		
REAGENT or RESOURCE	SOURCE	IDENTIFIER
Anti-HA Agarose beads	Sigma	Cat#A2095
Recombinant His-tagged human ATG4B	Abcam	Cat#ab188707
Deposited data		
https://data.mendeley.com/datasets/f5kjfmnf2p/1	N/A	N/A
Experimental models: cell lines		
HCT116 GFP-rLC3B	Fletcher et al., 2018	N/A
HCT116 *ATG16L1*−/− GFP-rLC3B	Fletcher et al., 2018	N/A
HCT116 GFP-rLC3B WT clone A	Ulferts et al., 2020 (BioRxiV)	N/A
HCT116 GFP-rLC3B *ATG4D*−/−	Ulferts et al., 2020 (BioRxiV)	N/A
MCF10A GFP-hLC3A	Florey et al., 2011	N/A
MCF10A *ATG13*−/− GFP-hLC3A	Jacquin et al., 2017	N/A
MCF10A *ATG13*−/− GFP-hLC3B	This manuscript	N/A
MCF10A *ATG13*−/− GFP-hLC3C	This manuscript	N/A
MCF10A *ATG13*−/− GFP-hGABARAP	This manuscript	N/A
MCF10A *ATG13*−/− GFP-hGABARAPL1	This manuscript	N/A
MCF10A *ATG13*−/− GFP-hGABARAPL2	This manuscript	N/A
J774.1A GFP-hLC3A	Florey et al., 2011	N/A
RAW264.7 GFP-hLC3A	This manuscript	N/A
RAW264.7 *ATG16L1*−/−	Lystad et al., 2019	N/A
RAW264.7 *ATG16L1*−/− GFP-hLC3A + WT FlagS-ATG16L1	This manuscript	N/A
RAW264.7 *ATG16L1*−/− GFP-hLC3A + K490A FlagS-ATG16L1	This manuscript	N/A
HeLa GFP-hLC3B.G120	Agrotis et al., 2019	N/A
HeLa ATG4B−/− GFP-hLC3B.G120	Agrotis et al., 2019	N/A
HEK293 FT	ATCC	ATCC Cat# PTA-5077, RRID:CVCL_6911
Oligonucleotides		
ATG4D guide 1	ggcgggacacaaagucccgc	N/A
ATG4D guide 2	gggacuuuguguccgccug	N/A
ATG4D guide 3	ccggcgguaugugagccac	N/A
Recombinant DNA		
pBabe-Puro GFP-GABARAP	MRC-PPU	DU36756
pBabe-Puro GFP-GABARAPL1	MRC-PPU	DU36757
pBabe-Puro GFP-GABARAPL2	MRC-PPU	DU40072
pBabe-Puro GFP-LC3B	MRC-PPU	DU40253
pBabe-Puro GFP-LC3C	MRC-PPU	DU40860
mRFP-Lact-C2	Addgene	Addgene plasmid Cat#74061

RESOURCE AVAILABILITY

Lead contact

Further information and requests for resources and reagents should be directed to and will be fulfilled by the Lead Contact (oliver.florey@babraham.ac.uk).

Materials availability

Plasmids and cell lines generated in this study will be made available upon request made to the Lead Contact (oliver.florey@babraham.ac.uk).

Data and code availability

Original imaging and western blots data were deposited at Mendeley at:
https://data.mendeley.com/datasets/f5kjfmnf2p/1

EXPERIMENTAL MODEL AND SUBJECT DETAILS

WT or *ATG13*$^{-/-}$ MCF10A cells (female, human breast epithelial), expressing GFP-LC3A (human), were prepared as described previously (Jacquin et al., 2017) and cultured in DMEM/F12 (GIBCO, 11320074) containing 5% horse serum (Sigma), EGF (20ng/ml; Peprotech AF-100-15), hydrocortisone (0.5 mg/ml; Sigma, H0888), cholera toxin (100 ng/ml; Sigma, C8052), insulin (10 μg/ml; Sigma, I9278), and penicillin/streptomycin (100 U/ml, /ml; GIBCO 15140-122) at 37°C, 5% CO_2. Briefly, wild-type and *ATG13*$^{-/-}$ cells generated by CRISPR/Cas9 using gRNAs (Fwd; TTTCTTGGCTTTATATATCTTGTGGAAAGGACGAAACACCGACAGCTGCCTG CAGTCGGG, Rev; GACTAGCCTTATTTTAACTTGCTATTTCTAGCTCTAAAACCCCGACTGCAGGCAGCTGTC), were transduced with pBabe-Blast hGFP-LC3A retrovirus as described below. These parental cell lines were also engineered to express alternative GFP-tagged isoforms of human ATG8s, using retroviral infection (pBabe-Puro) and antibiotic selection (2.5 μg/ml Puromycin).

HCT116 cells (male, human colorectal epithelial) expressing GFP-LC3B (rat) are an established model for CASM, used previously to study ATG16L1 mechanisms and Influenza A infection (Fletcher et al., 2018). These cells were maintained using DMEM (GIBCO, 41966-029) supplemented with 10% FBS (Sigma) and penicillin/streptomycin (100 U/ml, 100 μg/ml; GIBCO 15140-122) at 37°C, 5% CO_2. A panel of lines expressing different ATG16L1 constructs were derived from *ATG16L1*$^{-/-}$ cells, reconstituted with the pBabe-Puro ATG16L1 (wild-type or K490A), as described previously (Fletcher et al., 2018). Briefly, ATG16L1 was targeted in HCT116 cells with gRNA (ATTCTCTGCATTAAGCCGAT) designed to target exons shared by all predicted transcripts and cloned into the BpiI site of pSpCas9(BB)-2A-puro V2.0. Cells were transfected using Lipofectamine 2000 (Invitrogen) according to manufacturer's instructions and selected with puromycin (4 μg/ml), and single cell clones generated. ATG16L1 was inserted into pBabe Flag-S retroviral vector using SalI cloning sites. Alanine point mutants were generated using QuikChange Site-directed Mutagenesis Kit (Stratagene). Stable cell lines expressing ATG16L1 constructs were generated by retroviral transduction and selection as described below. ATG4D null cells were prepared as described below.

J774.A1 (female, mouse monocyte/macrophage) were obtained from ATCC and cultured in DMEM (GIBCO, 41966-029) supplemented with 10% FBS (Sigma) and penicillin/streptomycin (100 U/ml, 100 μg/ml; GIBCO 15140-122) at 37°C, 5% CO_2. These cells were engineered to express GFP-LC3A (human) by retroviral infection (pBabe-Blast) and antibiotic selection (8ug/ml Blasticidin), for use in LAP assays.

ATG16L1$^{-/-}$ RAW264.7 (male, mouse monocyte/macrophage) were described previously (Lystad et al., 2019) and cultured in DMEM (GIBCO, 41966-029) supplemented with 10% FBS (Sigma) and penicillin/streptomycin (100 U/ml, 100 μg/ml; GIBCO 15140-122) at 37°C, 5% CO_2. These cells were engineered to express GFP-LC3A (human, pBabe-Blast), and reconstituted with *ATG16L1* wild-type or K490A (pBabe-Puro), all by retroviral infection and selection (8 μg/ml Blasticidin, 2 μg/ml Puromycin), to assess the mechanisms of ATG16L1 during LAP.

HEK293FT cells (human, embryonic kidney) were grown in DMEM (GIBCO, 41966-029) supplemented with 10% FBS (Sigma) and penicillin/streptomycin (100 U/ml, 100 μg/ml; GIBCO 15140-122) at 37°C, 5% CO_2.

HeLa cells (female, human cervical adenicarcinoma epithelial) were cultured in DMEM (GIBCO, 41966-029) supplemented with 10% FBS (Sigma) and penicillin/streptomycin (100 U/ml, 100 μg/ml; GIBCO 15140-122) at 37°C, 5% CO_2. Wild-type and endogenously HA-tagged GABARAPL2 HeLa cells were kindly provided by Dr Christian Behrends (Eck et al., 2020). Wild-type and *ATG4B*−/− HeLa cells expressing GFP-hLC3B.G120 were kindly provided by Dr Robin Ketteler (Agrotis et al., 2019).

METHOD DETAILS

Reagents

Bafilomycin A1 (#1334) and PP242 (#4257) were purchased from Tocris; Monensin (M5273), DAPI (D9542) and human IgG (I4506) were from Sigma. GFP-Trap (gtma-20) and control magnetic agarose beads (bmab-20) were obtained from Chromotek, anti-HA agarose beads from Sigma (A2095), Magnetic 3-micron beads (PMA3N) from Bangs Laboratories and Latex polymer 3-micron beads (17134-15) from Polysciences. Murine IFNγ (315-05) was from Peprotech. Lipids were purchased from Avanti Polar Lipids (Alabaster, AL), dissolved in chloroform: 1,2-dioleoyl-sn-glycero-3-phosphoethanolamine (DOPE; 850725C), 1,2-dioleoyl-*sn*-glycero-3-phosphoethanolamine–rhodamine (DOPE–rhodamine; 810150C), 1-palmitoyl-2-oleoyl-sn-glycero-3-phosphocholine (POPC; 850457C) and 1,2-dioleoyl-sn-glycero-3-phospho-L-serine (DOPS; 840035C).

Plasmids

GFP-tagged, human LC3B, LC3C, GABARAP, GABARAPL1 and GABARAPL2, in pBabe-Puro, were purchased from MRC-PPU, University of Dundee. mRFP-Lact-C2 was a gift from Sergio Grinstein (Addgene plasmid # 74061). GFP-huLC3A pBabe-Blast was kindly provided by Dr Michael Overholtzer (MSKCC). Flag-S-tagged versions of mouse ATG16L1 (wild-type and K490A mutant), in pBabe-Puro, were previously described (Fletcher et al., 2018).

Generation of ATG4D CRISPR knock out cells

Stable ATG4D knock out cell lines were generated using CRISPR technology. HCT116 rGFP-LC3B cells were nucleofected with a pool of *in vitro* synthesized guide RNAs (Synthego) and Cas9 (Thermo). Single cell clones were isolated and absence of gene expression confirmed by western blotting. The sgRNAs were designed using the Synthego software: ATG4D guide 1: ggcgggacacaaa gucccgc, ATG4D guide 2: gggacuuuguguccccgccug, ATG4D guide 3: cccggcgguaugugagccac.

Retrovirus production and infection

Retrovirus production and infection was performed as described previously (Durgan et al., 2017). In brief, HEK293T cells were transfected with retroviral constructs and envelope and packaging constructs, using Lipofectamine 2000 (Invitrogen). Viral supernatant was collected over 2 days. For infection, cells were seeded in a 6 well plate at 5×10^4 per well. The next day 1ml viral supernatant was added with 10μg/ml polybrene for 24 hours followed by a media change. Selection was achieved with antibiotic treatment for 2-5 days. Constructs, plasmids and antibiotic concentrations are all indicated above.

Pharmacological stimulation

To induce canonical autophagy, cells were pretreated for 20 mins with 100 nM bafilomycin, followed by addition of 1 μM PP242 for a further 40mins. To induce non-canonical autophagy/CASM, cells were treated with 100 μM monensin for 60 mins (note: monesin also blocks autophagic flux in a WT genetic background). Stimulated cells were analyzed by microscopy, or lysed for western blotting or mass spectrometric analysis, as indicated.

J774.A1 phagosome preparation and assay

To induce, enrich and analyze phagosomes, J774.A1 cells expressing GFP-LC3A (human) were assayed with IgG coated magnetic beads (ProMag 3 Series-Amine, Bangs Laboratories). The magnetic beads were prepared according to the manufacture's guidelines. Briefly, beads were: i) washed in PBS and activated by rotating with 10% glutaraldehyde for 1 hour, RT; ii) washed in PBS and resuspended by rotating with 6 mg human IgG (Sigma, I4506) for 2 hours, RT; iii) washed again and quenched by rotating with 40 mM glycine for 1 hour, RT and iv) finally resuspended in PBS.

To enrich phagosomes for LC3 lipidation analysis, 8 × 15cm plates of J774.A1 cells were seeded per condition, incubated for 3 days, then stimulated with 200 U/ml murine IFNγ (Peprotech, 315-05) for 24 hours. Cells were then preincubated with 100 nM Bafilomycin A1, or DMSO control, for 15 mins. Phagocytosis was induced by adding IgG coated beads, which were incubated for 25 mins, 37°C. Cells were then placed on ice and washed with ice cold PBS. Each dish was scraped into 0.5 mL HB buffer: 250 mM sucrose, 10 mM HEPES, phosphatase inhibitors (1x, Sigma P0044) and protease inhibitors (1x, Sigma P8340), then spun at 200 rcm, 5 mins. The pellet (containing intact cells and beads) was resuspended in 1 mL fresh HB buffer and an aliquot of total cell extract removed. Cells were then gently ruptured with 35 strokes of a Dounce homogenizer, on ice. Samples were placed on a magnetic rack, to isolate the magnetic beads and their enclosing phagosomes. The beads were washed with 2x 1 mL HB buffer and parallel samples for each condition pooled; an aliquot of this phagosome preparation was withheld. Finally, to release and recover the phagosomal GFP-LC3 for analysis, the bead pellet was lysed in NP40 lysis buffer and subjected to GFP-TRAP IP, as described below.

RAW264.7 phagocytosis assay

RAW264.7 macrophage are an established model to study ATG16L1 during LAP (Lystad et al., 2019). IgG-coated latex beads were prepared as previously described (Jacquin et al., 2019). Briefly, 3-micron beads (Polysciences Inc) were resuspended in 0.1 M Borate and incubated with human IgG at 4°C overnight while rotating. The beads were washed in PBS x3, then resuspended in PBS. Opsinized zymosan was prepared by mixing zymosan with human serum for 30 mins at 37°C followed by washing and resuspension in PBS. RAW264.7 cells were seeded in 15 cm^2 dishes and treated with 200 U/ml IFNγ (Peprotech, 315-05) for 24 hours prior to use. Where indicated, 350 ul IgG beads, or 175 ul zymosan (10mg/ml), were added to dishes for 30 minutes at 37°C. Cells were washed in cold PBS x 1 and lysed in 900 ul lysis buffer consisting of: 50 mM Tris pH 7.5, 150 mM NaCl, 0.5% NP40 (IGEPAL CA-630, Sigma I3021), phosphatase inhibitors (1x, Sigma P0044) and protease inhibitors (1x, Sigma P8340). Samples were scraped into pre-chilled 1.5 mL Eppendorf tubes, incubated on ice for 20 minutes and centrifuged at 13,500 rpm for 10 minutes at 4°C. Notably, induction of LAP was so robust and specific under these conditions, that phagosome enrichment was not necessary. The supernatants were subjected directly to GFP-TRAP IP, as described below.

Influenza A infection

Stocks of influenza A virus PR8 (strain A/Puerto Rico/8/1934) were generated using an eight plasmid-based system, as previously described, (de Wit et al., 2004), and propagated on MDCK cells. In brief, eight genomic segments from influenza virus A/PR/8/34 were amplified by RT-PCR and cloned in pSP72-PhuThep (segments 2 and 6) or pSP72-PhuTmu (all other segments). The constructs were then transfected into 293T cells together with expression plasmids for the polymerase proteins and nucleoprotein of influenza virus A/PR/8/34: HMG-PB2, HMG-PB1, HMG-PA, and HMG-NP, using transient calcium phosphate-mediated transfection. At 72 hours post transfection, supernatants were harvested and virus titrated.

For infection, cells were washed with serum-free DMEM, then incubated with virus in serum-free DMEM at 37°C. After 1 h, the medium was replaced with DMEM containing 10% FBS. Cells were processed 16 hours post infection (h.p.i.) and analyzed by microscopy, or lysed for western blotting or mass spectrometric analysis, as indicated.

Whole cell lipidomic analysis

5×10^5 MCF10A cells were seeded per 6 cm dish, incubated overnight and then treated as indicated. These cells adhere strongly and tend to rupture upon scraping, so were harvested by trypsinsation. Cells were rinsed in PBS, incubated with trypsin for 3 minutes, 37°C and harvested in media. The cells were then washed 3x in PBS and pelleted at 150 *rcf.*, 3 minutes. Cell pellets were snap frozen in liquid nitrogen for lipid analysis. The frozen cell pellets were subjected to Folch extraction using chloroform/MeOH/H_2O (2:1:1). The dry extract was re-suspended in chloroform/MeOH (1:1). Phosphatidylethanolamine (PE) and phosphatidylserine (PS) were separated by liquid chromatography using a Shimadzu XR system (Shimadzu, Kyoto, Japan) (Zhuang et al., 2019). PE and PS were then detected using an Orbitrap Elite mass spectrometer in full scan mode with a mass range of 400- 1000 *m/z* at a target resolution of 240,000 (FWHM at *m/z* 400). Data were analyzed using Lipid Data Analyzer (2.6.0–2) software (Hartler et al., 2017).

Cell lysis and GFP-TRAP immunoprecipitation

Cells expressing GFP-ATG8 were seeded across multiple 15-cm dishes, treated as indicated, then placed on ice and washed with ice-cold PBS. Each 15-cm dish was scraped into 900 μL lysis buffer. Lysis composition was as follows: 1) GFP-LC3A/B in MCF10A/RAW264.7: 50 mM Tris pH 7.5, 150 mM NaCl, 0.5% NP40 (IGEPAL CA-630, Sigma I3021), phosphatase inhibitors (1x, Sigma P0044) and protease inhibitors (1x, Sigma P8340); 2) GFP-LC3B in HCT116 cells, as above, except 1% Triton replaces 0.5% NP40; 3) GFP-GABARAPs in MCF10A: as above, but with the addition of 20 mM N-Ethylmaleimide (NEM) to protect the lipidated species (Agrotis et al., 2019). The resulting suspension was incubated on ice for 10 minutes, then centrifuged at 16000 rcm, 4°C, 10 minutes to separate the pellet from the soluble lysate. A small fraction of the supernatant was removed for western blotting, as described below, and the remaining lysate subjected to preclearing and IP, using magnetic beads (Chromotek) and a magnetic separation rack (Cell Signaling), according to the manufacturers' instructions. The lysate was pre-cleared, using 10 μL equilibrated magnetic agarose control beads/sample (bmab, Chromotek), for 30 minutes, 4°C, on a rotating wheel. Cleared lysates were then incubated with 10 μL equilibrated GFP-TRAP beads/sample (gtma, Chromotek) for 60 minutes, 4°C, on a rotating wheel, to recover GFP-LC3. The beads were washed 3 × 10 minutes in lysis buffer at 4°C, on a rotating wheel. Enriched GFP-LC3 was either processed further on the beads (see ATG4B delipidation assay), or eluted for analysis by Mass Spectrometry with the addition of 25 μL 2x LDS buffer (Invitrogen)/0.2 M DTT sample buffer at 100°C, 5 minutes.

HA-immunoprecipitation

Wild-type and HeLa cells expressing endogenously HA-tagged GABARAPL2 were each seeded across 5 × 15 cm^2 dishes per condition. Cells were treated as indicated, then placed on ice and washed with ice-cold PBS. Each 15-cm^2 dish was scraped into 900 μL lysis buffer: 50 mM Tris pH 7.5, 150 mM NaCl, 1% Triton, phosphatase inhibitors (1x, Sigma P0044) and protease inhibitors (1x, Sigma P8340). The resulting suspension was incubated on ice for 10 minutes, then centrifuged at 16000 rcm, 4°C, 10 minutes to separate the pellet from the soluble lysate. A small fraction of the supernatant was removed for western blotting, as described below, and the remaining lysate subjected to IP using 100 ul equilibrated anti-HA agarose beads/sample for 60 minutes at 4°C on a rotating wheel. Enriched HA-GABARAPL2 was eluted for analysis by Mass Spectrometry with the addition of 25 μL 2x LDS buffer (Invitrogen)/0.2 M DTT sample buffer at 100°C, 5 minutes.

Mass spectrometric analysis of lipidated ATG8

ATG8 samples were run on 10% NuPAGE gels in MOPS buffer (Invitrogen), alongside protein molecular weight markers (EZ-Run, Fisher). Gels were released into a MeOH rinsed box for washing and staining, all at RT, with gentle shaking. Each gel was washed 3 × 5 mins in dH2O, stained with Imperial Stain (Thermo Scientific, 24615) for 2 hr, then destained in dH2O overnight. Stained gels were scanned and representative images are presented. For each sample, the entire gel region, containing both lipidated and non-lipidated ATG8 protein, was excised into a single tube, destained, and typically saponified by treatment with 50 mM NaOH in 30% MeOH at 40°C for 2 hr. The protein was digested with AspN protease (Roche) at 30°C for 16 hr, in 25 mM ammonium bicarbonate, which cleaves predominantly to the N-terminal side of Asp residues.

For the initial characterization of modified LC3A, peptides were separated on a reversed-phase nanoLC column (150 × 0.075mm; Reprosil-Pur C18AQ, Dr Maisch), interfaced to an Orbitrap Velos Pro mass spectrometer (Thermo Scientific), operating in high resolution (orbitrap) MS1 mode, with data-dependent acquisition of low resolution MS2 spectra generated by CID in the linear ion-trap. The measured neutral monoisotopic masses of the three forms of LC3A C-terminal peptide DGFLYMVYASQETFG, calculated from the predominant doubly protonated pseudomolecular ions, were: unmodified - 1726.758 (theoretical 1726.754); PE-modified - 1923.802 (theoretical 1923.800); PS-modified - 1967.790 (theoretical 1967.789). MS2 data were searched against the Uniprot mouse proteome database using Mascot software (Matrix Science), with glycerophosphoethanolamine and glycerophosphoserine combined with loss of the C-terminal amino-acid, specified as custom C-terminal variable modifications. Spectral matches to the C-terminal modified peptides were confirmed by manual interpretation. Some y-ions gave a secondary fragment consistent with neutral loss of phosphoglycerol (172). As expected, b-ions did not shift. b14 is characteristically absent from the unmodified peptide, but

observed in the modified peptides, along with b* (cleavage between Gly and head group), confirming C-terminal amide linked modification.

It was observed during the characterization of the C-terminal peptides that the Met residue was > 90% oxidized to the sulphone, so in order to increase the sensitivity of subsequent targeted analyses, the Met-oxidised forms of the peptides were used. For the targeted mass spectrometric assay of C-terminal ATG8 peptides, samples were processed identically, but the analysis was done on a Q-Exactive mass spectrometer (Thermo Scientific). The hLC3B, hLC3C, GABARAP, GABARAPL1 and GABARAPL2 peptides, and their neutral monoisotopic masses, are shown in Figure S1A. For each of the ATG8 protein analyses, the mass spectrometer scan cycle consisted of one high-resolution MS1 scan, and three high resolution MS2 scans from fragmentationof the parent ions of the unmodified, glycerophosphoethanolamine- and glycerophosphoserine-modified C-terminal peptides.

Quantitative data were extracted using Skyline software (MacCoss Lab, University of Washington) using the sum of the chromatographic peak areas from the y-series fragment ions. Normalization was performed against unmodified C-terminal peptides.

On bead ATG4B delipidation assay

ATG13$^{-/-}$ MCF10A cells were treated -/+ 100 μM monensin for 60 minutes and GFP-hLC3A was enriched, immobilised and washed on GFP-TRAP beads, as described above. Recombinant His-tagged human ATG4B (Abcam, ab188707) was pre-treated with 10 mM DTT for 15 mins, RT, to achieve maximum activation, then added at 2 μg/sample, in lysis buffer, to the GFP-LC3 beads for 0-120 minutes at 37°C. At the end of the time-course, the reaction mixture was aspirated, the beads quickly rinsed with ice cold lysis buffer and GFP-hLC3A was eluted for analysis by Mass Spectrometry with 25 μL 2x LDS (Invitrogen)/0.2 M DTT sample buffer at 100°C, 5 mins.

Protein purification for liposome assays

Full-length ATG5–12–ATG16L1 complex was expressed and purified from HEK suspension cells (HEK-F, Invitrogen) in 200 mL scale. Cells were grown to 2–3 × 10^6 cells/ml on a shaker (160 rpm) at 37°C with 8% CO_2 in 4 mM glutamine supplemented BalanCD medium (Irvine Scientific). A total of 1 μg per 1 × 10^6 cells of the following plasmids was mixed with a threefold excess (w/w) of polyethyleneimine “MAX” (40 kDa, Polysciences, Inc.) in 8 mL OptiPro (Invitrogen): pCMV-3xFLAG-SUMOstar-hATG16L1, pCMV-hATG5, pCMV-GST-hATG12, in amount ratio 1:2:2. To this was added 10% (w/w) of plasmid pCMV-hATG10, and the mixture was incubated for 20 min at room temperature before added to the cells. Cells were grown for three days with addition of 5% BalanCD Feed (Irvine Scientific) after 1 and 2 days. Cells were centrifuged at 350 × g for 5 min, washed with 30 mL PBS, and the cell pellet was lysed with 22 mL PBS containing 1% Nonidet P40 (Pierce), 1 mM EDTA, and cOmplete ULTRA protease inhibitors (Roche). After 15 min incubation on ice, lysed cells were centrifuged at 350 × g for 5 min and the supernatant was collected, snap frozen in liquid nitrogen, and stored at −80°C.

Lysate was thawed and centrifuged at 20.000 × g for 10 min and the supernatant was added to 3 mL of anti-FLAG (M2)-agarose (Sigma), and incubated 5h in the cold by end-to-end rotation. The gel matrix was transferred to a column and washed stepwise by at least 5 column volumes of NT350 (350 mM NaCl, 20 mM Tris-HCl, pH 7.4). The gel was resuspended in 1 mL NT350 to which was added 2 μL SUMOstar protease (20 U, Life Sensors) and the closed column was incubated in the refrigerator overnight. Cleaved protein was eluted by stepwise 1 mL additions of NT350 and fractions with highest amount of protein were pooled and added to 0.5 mL of glutathione-Sepharose (GE Healthcare), equilibrated with NT350. After 5 h incubation by end-to-end rotation in the cold, the gel matrix was washed three times with 1 mL NT350 by centrifugation and resuspended in 1 mL NT350 with 20 μg GST-HRV 3C protease (produced in-house at 4 mg/ml by expression from a pGEX plasmid in *E. coli*). The gel was incubated at 4°C by end-to-end rotation overnight, pelleted and the cleaved complex was collected in the supernatant, snap-frozen in liquid nitrogen, and stored at −80°C.

Human ATG3, ATG7, ATG4A, ATG4B, ATG4C and ATG4D were expressed and purified from HEK suspension cells in a similar procedure to that described for ATG12-5-16L1, with the second purification step omitted.

LC3B (amino acids 1-120) and GABARAP (amino acid 1-116) were expressed in BL21 pLysS DE3 *E.coli* cells from a pGEX-6P-2 plasmid (GE Healthcare) in 250 mL LB medium. After induction with IPTG at OD 0.8, the cells were grown at 22°C for 4 hours and harvested by centrifugation. After washing with NH100 (100 mM NaCl, 20 mM HEPES-KOH pH 7.4), the cells were resuspended in 5 mL NH100 and snap-frozen. The cells were thawed and centrifuged at 75.000 x g for 30 minutes, and the supernatant was incubated with glutathione-Sepharose. After washing with NH100, LC3B was eluted by on-column cleavage overnight at 4°C with HRV 3C protease. Eluted protein was gel filtrated on Sephacryl S-200 HR (GE Healthcare) equilibrated with NH100 buffer, and purified LC3B was snap-frozen and stored at −80°C.

RavZ was expressed in BL21-Gold (DE3) *E. coli*. Cells were grown at 37°C to an OD of 0.6–0.8 before protein expression was induced with 0.5 mM IPTG. Cells were then grown for three additional hours before they were collected by centrifugation. Cells were resuspended in NT350, supplemented with a Roche Complete Protease Inhibitor, lysed by sonication, and cleared by centrifugation (20.000 x g for 10 minutes). The supernatant was incubated at 4°C with Glutathione Beads (Sigma) for 4 hours. Beads were collected and washed twice with NT350 buffer before HRV 3C protease was added and allowed to cut at 4°C overnight. The next morning, protein fractions were collected and stored at −80°C.

Liposome assays

To prepare liposomes the various lipid combinations were dried under nitrogen gas, and the lipid film was further dried under vacuum for 1 hour. The lipids were reconstituted in NT350 buffer (350 mM NaCl, 20 mM Tris–HCl pH 7.4) and subjected to seven cycles of

CellPress
OPEN ACCESS

flash-freezing in liquid nitrogen and thawing in a 37°C bath. PE liposomes were composed of 50 mol% POPC and 50 mol% DOPE, PS liposomes contained 50 mol% POPC and 50 mol% DOPS, while the mixed liposomes were composed of 19.9 mol% POPC, 40 mol% DOPE, 40 mol% DOPS and 0.1 mol% DOPE–rhodamine. Liposomes were further sonicated immediately prior to the lipidation reaction.

Lipidation reactions were carried out in microcentrifuge tubes containing ATG7 (0.5 μM), ATG3 (1 μM), ATG12-ATG5-ATG16L1 (0.1 μM), LC3B (aa1-120, 10 μM) or GABARAP (aa1-116, 10μM) and sonicated liposomes (3 mM), mixed in NT350 buffer containing 1 mM DTT.

Lipidation was initiated by adding 1 mM ATP and reactions were incubated at 37°C for 90 minutes. The lipidation reaction was then run on a Nycodenz density gradient to remove non-lipidated LC3B/GABARAP from the proteoliposomes. The bottom layer of the gradient consisted of 150 μL of 80% Nycodenz and 150 μL of the lipidation reaction. The second layer consisted of 250 μL of 30% Nycodenz while the top layer was 50 μL of NT350 buffer. Gradients were spun at 48000 rpm at 4°C for 4 hours in a Beckman SW55Ti rotor. Liposomes with lipidated LC3B/GABARAP were collected from the top of the tube and stored at 4°C, before use in subsequent de-lipidation experiments.

To measure de-lipidation of LC3B-PE/LC3B-PS/GABARAP-PE/GABARAP-PS, proteoliposomes (~1 μM LC3B-II) were mixed with NT350 containing 1 mM DTT and kept on ice until activity assays were initiated by addition of various ATG4 proteins (0.5 μM) or RavZ (0.5 μM). Reactions were incubated at 30°C for 1 hour, before they were mixed with sample buffer and immediately boiled to stop proteolysis. The samples were then separated using SDS-PAGE, visualized by Coomassie staining and either analyzed directly with ImageLab (Biorad) to assess bandshift, or processed for Mass Spectrometry, as described above. To quantify levels of delipidation, densitometry LC3B-I, LC3B-II, GABARAP-I and GABARAP-II in Coomassie images was performed using ImageJ.

Western blotting

Western blotting was performed as described previously (Fletcher et al., 2018; Jacquin et al., 2017). Briefly, cell lysates were run on SDS-PAGE gels (10%, 12% or 15%), transferred to PVDF membrane (Immobilon-P, Millipore), blocked with 5% BSA (Sigma A7906)/TBS-T for 1 hour, RT and then incubated with primary antibody at 4°C overnight. The following antibodies were used, all at 1:1000: anti-ATG4B (Cell Signaling, 5299), anti-ATG4D (Proteintech, 16924-1-AP), anti-ATG13 (Cell Signaling, 13468), anti-ATG16L1 (Cell Signaling, 8089), anti-GAPDH (Abcam, ab9843), anti-GFP (Sigma, 11814460001), anti-HA (Roche, 11867423001) and anti-M2 (Abcam, ab5416). Membranes were washed 3x 10 minutes in TBS-T and incubated with HRP-conjugated secondary antibodies (Cell Signaling 7074, 7076) for 45 minutes, RT. Membranes were washed again 3x 10 minutes in TBS-T, then developed with ECL (GE, RPN2209). Blots were scanned (Epson Perfection, V550) and images are representative of 3 separate experiments.

Microscopy

For fixed cell imaging, cells were rinsed in PBS, then fixed in cold methanol at −20°C for 5 minutes. Samples were washed with PBS and blocked in 5% BSA/PBS for 1 hour at room temperature. Where indicated, primary antibodies were diluted in blocking buffer and added overnight at 4°C. Cells were washed and secondary antibody, diluted in blocking buffer, added for 1 hour. Final washes were performed, before incubating with DAPI for 10 minutes and mounting in ProLong Gold Antifade (ThermoFisher, P36930). Samples were analyzed on a Confocal Zeiss LSM 780 microscope (Carl Zeiss Ltd), equipped with a 40x oil immersion 1.40 numerical aperture (NA) objective using Zen software (Carl Zeiss Ltd).

For live cell imaging of Lact-C2-RFP during autophagosome formation, cells were grown on 35-mm MatTek glass bottomed dishes. Imaging was performed within an incubation chamber at 37°C and 5% CO_2, with Z stacks acquired every 20 s using a spinning disk confocal microscope, comprising a Nikon Ti-E stand, Nikon 60x 1.45 NA oil immersion lens, Yokogawa CSU-X scanhead, Andor iXon 897 EM-CCD camera and Andor laser combiner. Image acquisition and analysis was performed with Andor iQ3 (Andor Technology, UK) and ImageJ.

For Lact-C2-RFP analysis during CASM, z stacks were taken using a Confocal Zeiss LSM 780 microscope (Carl Zeiss Ltd) equipped with an environment chamber, at 37°C and 5% CO_2, and a 40x oil immersion 1.40 numerical aperture (NA) objective using Zen software (Carl Zeiss Ltd).

For live imaging and quantification of phagocytosis, cells were grown on 35-mm MatTek glass bottomed dishes and z stacks acquired using a Confocal Zeiss LSM 780 microscope (Carl Zeiss Ltd) equipped with a 40x oil immersion 1.40 numerical aperture (NA) objective using Zen software (Carl Zeiss Ltd). Samples were imaged and maintained in an environment chamber at 37°C and 5% CO_2. For phagocytosis quantification, CellMask (ThermoFisher, C10046) was added to cells prior to imaging. Phagocytosis was quantified as the number of phagosomes per cell that were CellMask negative. Image analysis was performed using ImageJ software.

LC3B-ATG4 complex modeling

The following X-ray crystal structures were used for making models used in illustrations: ATG4B-LC3B 1-120 complex (human), PDB: 2z0e (1.9 Å; Rfree 0.23) (Satoo et al., 2009), ATG4B (human), PDB: 2cy7 (1.9 Å; Rfree 0.25) (Sugawara et al., 2005), PE head group, PDB: 3PE from PDB: 6tzk (1.8 Å; Rfree 0.18) (Acheson et al., 2019), PS head group, PSF.pdb from PDB: 3bib (2.5 Å; Rfree 0.25) (Santiago et al., 2007). The model of LC3B-PE was made by superimposing the ethanolamine moiety of PE (PDB: 3PE) on the backbone (Cα), side chain (Cβ) and N of THR121 of LC3B (PDB: 2z0e), with an amide bond built between the ethanolamine N and C-terminal

carboxyl of GLY120 of LC3B (PDB: 2z0e) using PyMOL (The PyMOL Molecular Graphics System, Version 2.2, Schrödinger, LLC). The phosphate of the PE head group was positioned to avoid clashes with ATG4B atoms (PDB: 2z0e) by rotation about the ethanolamine carbons. The model of LC3B-PS was made in the same manner using the head group of PS (PDB: 3PSF). The ATG4B-LC3B structure contains an HIS280ALA mutation to facilitate stable complex formation. The ALA280 of PDB; 2z0e was changed to a HIS, and its position modeled on that found in the native structure of ATG4B (PDB: 2cy7), in order to illustrate the active site of ATG4B.

QUANTIFICATION AND STATISTICAL ANALYSIS

Two-tailed Ratio-paired t tests, or Student's t tests, were performed using Graph Pad, as indicated. Information on number of repeats is included in the relevant figure legend.

Molecular Cell, Volume *81*

Supplemental information

Non-canonical autophagy drives alternative ATG8 conjugation to phosphatidylserine

Joanne Durgan, Alf H. Lystad, Katherine Sloan, Sven R. Carlsson, Michael I. Wilson, Elena Marcassa, Rachel Ulferts, Judith Webster, Andrea F. Lopez-Clavijo, Michael J. Wakelam, Rupert Beale, Anne Simonsen, David Oxley, and Oliver Florey

Supplemental Figure S1

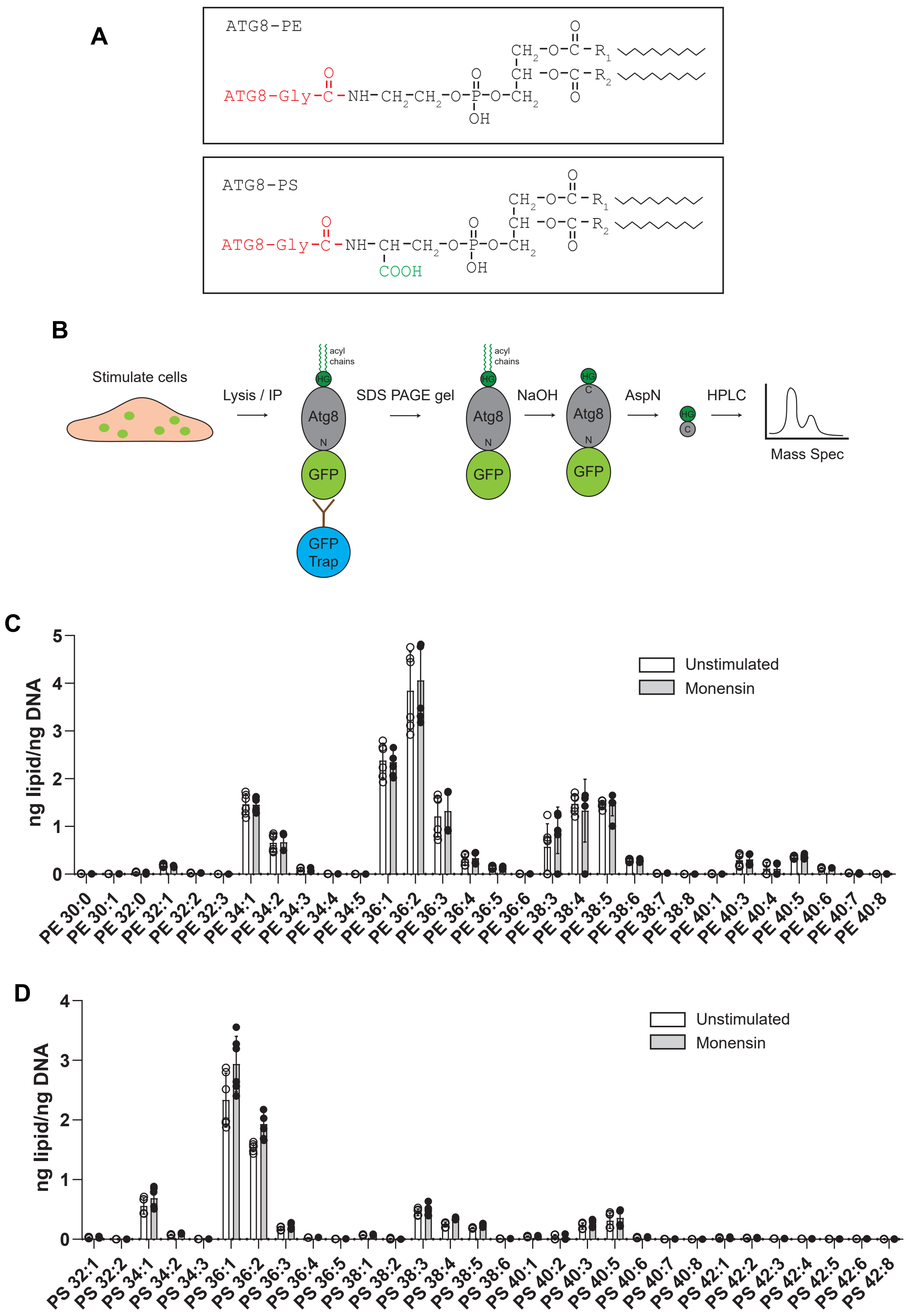

Figure S1. Analysis of ATG8 lipidation and PE/PS lipidomics, Related to Figure 1 and STAR Methods.

(A) Schematics representing ATG8 conjugated to PE and PS.
(B) Work flow for GFP-ATG8 mass spectrometry analysis. Cells are stimulated as indicated, then subjected to lysis and GFP-ATG8 immunoprecipitation using GFP-TRAP beads. Samples are run on SDS-PAGE gels, coomassie stained and appropriate bands excised and base treated (NaOH) to strip lipid acyl chains. HG = lipid headgroup. Samples are then digested using AspN protease, separated by HPLC and analysed by mass spectrometry.
(C and D) Global lipids quantification from *ATG13*$^{-/-}$ MCF10A cells -/+ 100 μM monensin, for 60 mins, were analysed for phosphoethanolamine (PE) (C) or phosphatidylserine (PS) (D) molecular species. Data represent means from 6 biological replicates ± SD, with no major changes.

Supplemental Figure S2

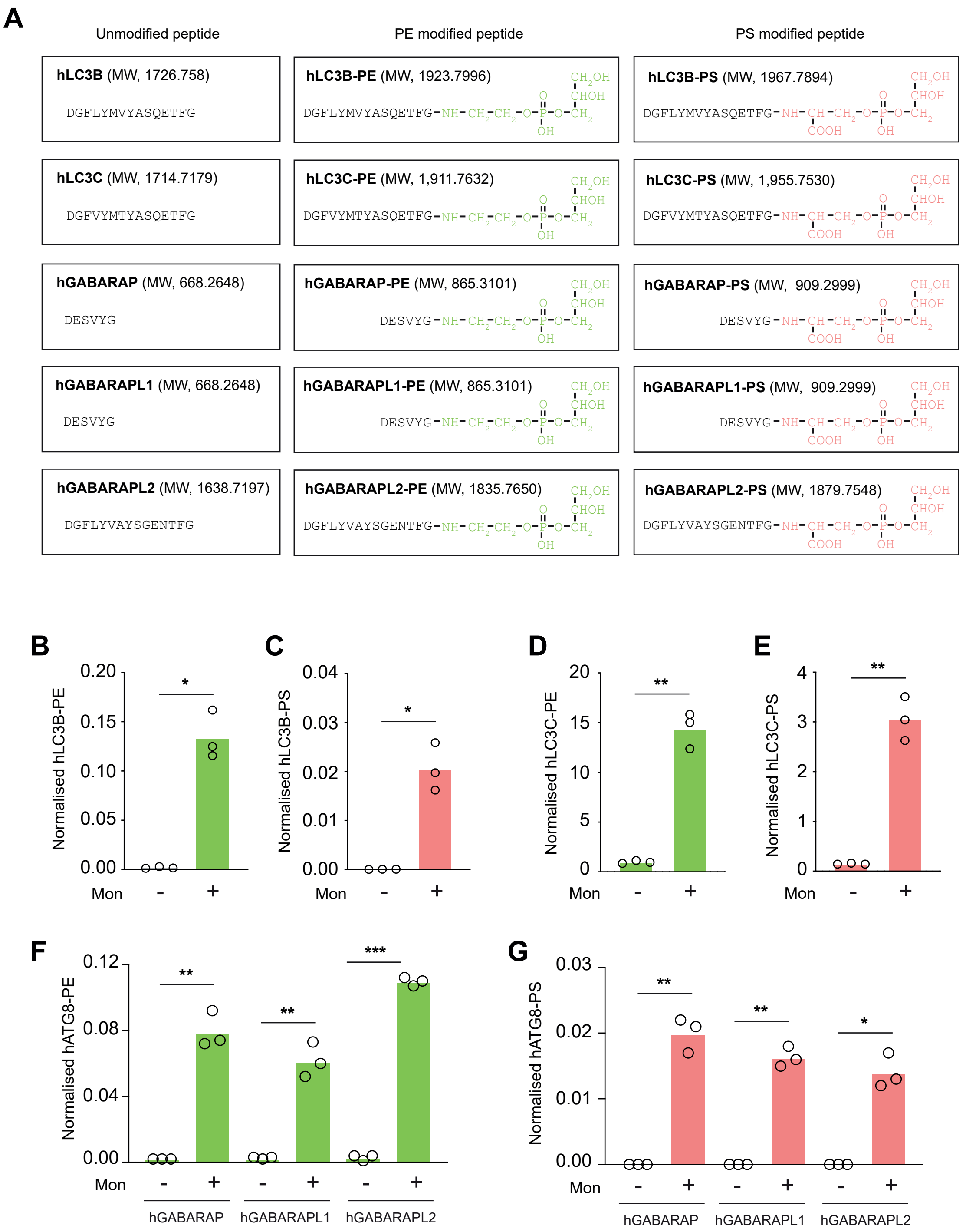

Figure S2. All human ATG8 proteins are conjugated to PS during non-canonical autophagy, Related to Figure 1.

(A) Schematics representing C-terminal peptides of human LC3B, LC3C, GABARAP, GABARAPL1 and GABARAPL2 proteins conjugated to either the PE or PS headgroup. Predicted MWs are indicated.

(B – G) Normalised mass spectrometry analysis of GFP-tagged human ATG8 proteins from monensin treated MCF10A *ATG13*$^{-/-}$ cells.

Data represent means from 3 independent experiments, ***$p<0.0002$, **$p<0.002$, *$p<0.03$, paired t-test.

Supplemental Figure S3

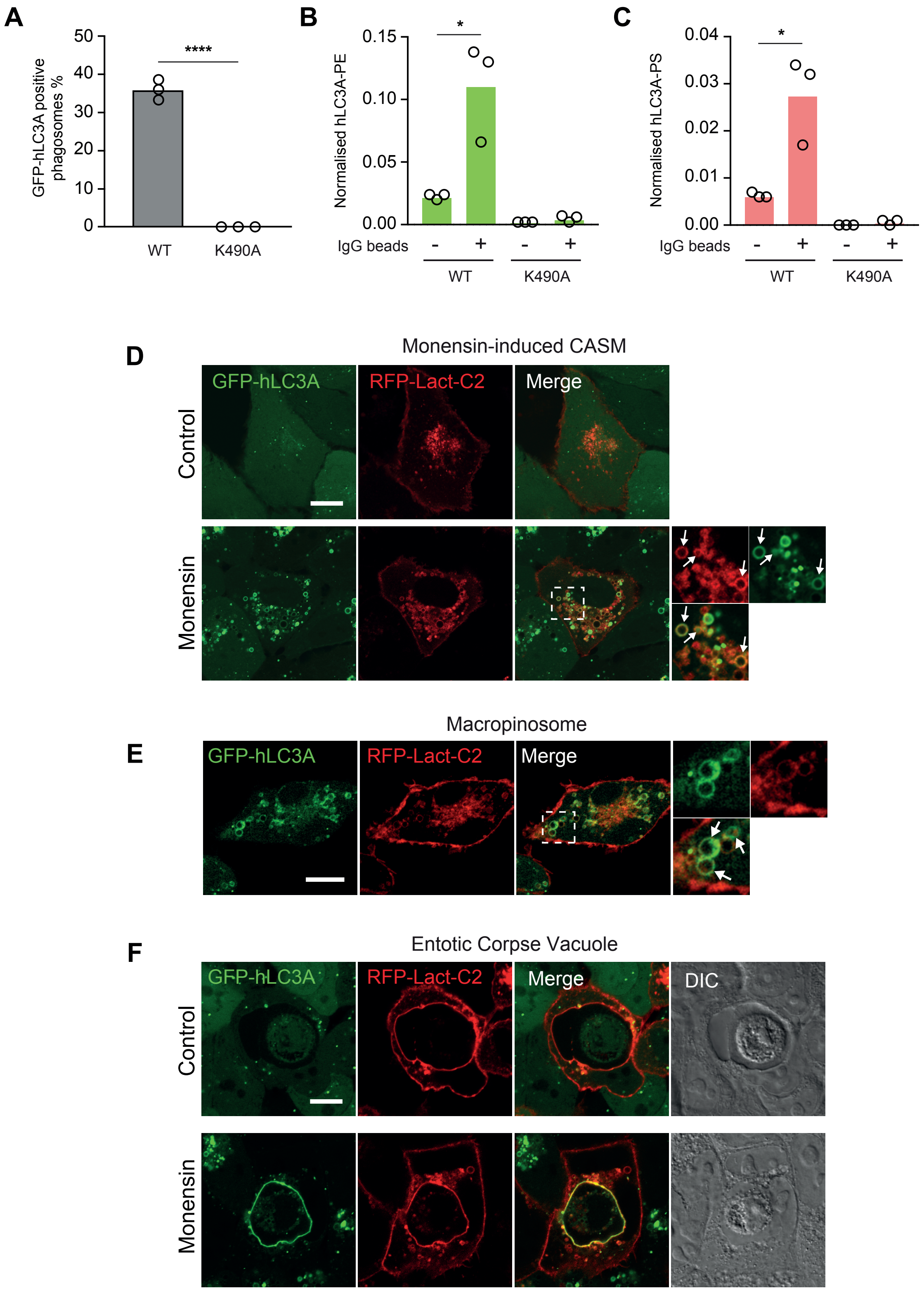

Figure S3. ATG8-PS conjugation depends on the ATG16L1 WD40 domain during LAP, and GFP-LC3A is recruited to PS positive membranes during CASM, Related to Figure 3.

(A) Quantification of GFP-hLC3A positive IgG-bead containing phagosomes in RAW264.7 ATG16L1-/- cells re-expressing WT or K490A ATG16L1. Data represent means from 3 independent experiments, WT n=189, K490A n=150 phagosomes in total, ****$p<0.0001$, unpaired t-test.
(B and C) Normalised mass spectrometry analysis of GFP-hLC3A conjugated to PE or PS during LAP in RAW264.7 cells expressing ATG16L1 WT or K490A. Data represent means from 3 independent experiments, *$p<0.01$, unpaired t-test.
(D) Confocal images of MCF10A expressing GFP-hLC3A and RFP-Lact-C2 +/- monensin treatment for 40 mins. Dashed box indicates zoomed region. Arrows indicate double-positive membranes. Scale bar: 10 μm.
(E) Confocal images of J774A.1 cells expressing GFP-hLC3A and PS sensor RFP-Lact-C2. Arrows denote macropinosomes. Scale bar: 5 μm.
(F) Confocal images of entotic corpse vacuoles in MCF10A expressing GFP-hLC3A and RFP-Lact-C2 +/- monensin treatment. Scale bar: 10 μm.

Supplementary Figure S4

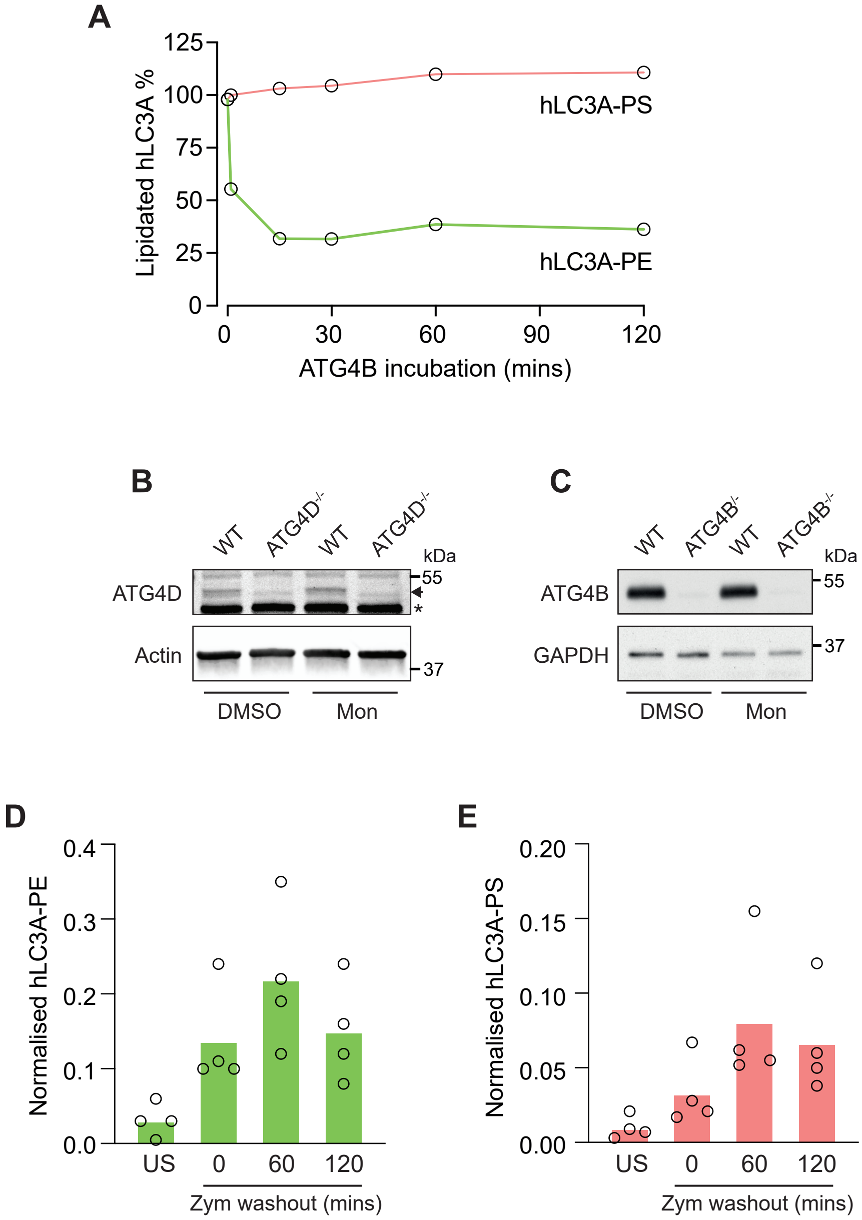

Figure S4. ATG4 delipidation of ATG8-PE and ATG8-PS, Related to Figure 4.

(A) Mass spectrometry analysis of GFP-hLC3A immunoprecipitated from MCF10A cells treated with monensin followed by incubation with recombinant ATG4B for the indicated times. Data are normalised to time 0.

(B and C) Western blot analysis of wild type and *ATG4D*$^{-/-}$ HCT116 cells (B) and wild type and *ATG4B*$^{-/-}$ HeLa cells (C) treated -/+ monensin. Arrow indicates ATG4D, asterisk indicates non-specific band.

(D and E) Normalized mass spectrometry analysis of GFP-hLC3A-PE and GFP-hLC3A-PS in RAW264.7 cells stimulated with zymosan for 25 mins followed by washout for the indicated times. Data represent means of 4 independent experiments.
